# A review of *Crassignatha* (Araneae, Symphytognathidae)

**DOI:** 10.3897/zookeys.988.56188

**Published:** 2020-11-06

**Authors:** Ya Li, Yucheng Lin, Shuqiang Li

**Affiliations:** 1 Key Laboratory of Bio-resources and Eco-environment (Ministry of Education), College of Life Sciences, Sichuan University, Chengdu, Sichuan 610064, China; 2 Institute of Zoology, Chinese Academy of Sciences, Beijing 100101, China; 3 The Sichuan Key Laboratory for Conservation Biology of Endangered Wildlife, Sichuan University, Chengdu, Sichuan 610064, China

**Keywords:** Asia, barcode, new combination, new species, redescription, symphytognathids, taxonomy

## Abstract

*Crassignatha* Wunderlich, 1995 is redefined to include species with six eyes in three diads, chelicerae fused only near the base, sculpturing on the carapace, one or two clasping spurs on tibia II, a bilateral scutum of the male abdomen, and globular spermathecae and adjacent copulatory openings in the female. A key and distribution map are provided for 24 *Crassignatha* species in this paper. Diagnoses and illustrated photographs are provided for 22 species from China, Malaysia, Thailand, and Vietnam. Thirteen species are described and documented as new to science: *C. baihua* Y. Lin & S. Li, **sp. nov.** (♂♀), *C. bangbie* Y. Lin & S. Li, **sp. nov.** (♀), *C. changyan* Y. Lin & S. Li, **sp. nov.** (♀), *C. dongnai* Y. Lin & S. Li, **sp. nov.** (♀), *C. gucheng* Y. Lin & S. Li, **sp. nov.** (♂♀), *C. mengla* Y. Lin & S. Li, **sp. nov.** (♂♀), *C. nantou* Y. Lin & S. Li, **sp. nov.** (♂♀), *C. nasalis* Y. Lin & S. Li, **sp. nov.** (♂♀), *C. rostriformis* Y. Lin & S. Li, **sp. nov.** (♂♀), *C. shunani* Y. Lin & S. Li, **sp. nov.** (♂♀), *C. si* Y. Lin & S. Li, **sp. nov.** (♂♀), *C. thamphra* Y. Lin & S. Li, **sp. nov.** (♀), and *C. xichou* Y. Lin & S. Li, **sp. nov.** (♀). Three new combinations are proposed: *C. bicorniventris* (Lin & Li, 2009), **comb. nov.**, *C. quadriventris* (Lin & Li, 2009), **comb. nov.**, and *C. shiluensis* (Lin & Li, 2009), **comb. nov.** are transferred from *Patu* Marples, 1951. DNA barcodes and genetic distances of seventeen species are obtained to confirm correct identification. Types of seven known Chinese *Crassignatha* species are re-examined, and the taxonomic placement of *C. longtou* Miller, Griswold & Yin, 2009 may be incorrect based on morphological and molecular data.

## Introduction

Symphytognathidae Hickman, 1931 is a category of super miniature (body size ca. 1 mm or less), poorly known, araneoid spiders that contains eight genera and 74 documented extant species (WSC 2020; Li 2020), but no fossil species have been recorded to date (Dunlop et al. 2020). With the exception of Antarctica, they are found on all continents and some oceanic islands, distributed in the tropical and subtropical regions. They are cryptozoic, commonly found in the leaf litter layer of forests, and some species inhabit dark caves (Cardoso and Scharff 2009; Lin and Li 2009; Miller et al. 2014).

*Crassignatha* Wunderlich, 1995 was erected as a monotypic genus and originally placed in Synaphridae Wunderlich, 1986. The phylogenetic analysis of Lopardo and Hormiga (2015) corroborated its placement within Symphytognathidae. Since its inception, the genus has included nine described species, which are only found in the Gaoligong Mountains of southwest China (Miller et al. 2009), Malaysia (Wunderlich, 1995; Miller et al. 2014). The distribution of *Crassignatha* is currently confined to Southern China and Southeast Asia. There has been no worldwide or regional taxonomic revision of the genus.

From 2006 to 2018, we have accumulated a considerable number of symphytognathid specimens during many field collecting trips in China, Vietnam, Thailand, Myanmar, and Indonesia. Some of these specimens were described as new species (Lin and Li 2009; Lin et al. 2009; Lin et al. 2013; Lin 2019). However, further morphological and molecular studies of this material have revealed an extraordinary species diversity in the aforementioned geographic areas.

The main aim of this paper is to provide a comprehensive overview of *Crassignatha* and to report 22 species which are found in Southern China and the Indo-China Peninsula. The circumscription and diagnosis of *Crassignatha* are reconfirmed. Thirteen species are described and designated as new members of *Crassignatha*, as well as three new combinations that are transferred from *Patu* Marples, 1951. The types of seven known *Crassignatha* species from the Gaoligong Mountains in Southwest China are re-examined. A key and distribution records are provided for all *Crassignatha* species.

## Materials and methods

Most of the specimens from this study were collected by hand or sifting leaf litter and immediately preserved in a 95% ethanol solution. Type specimens of other known Chinese *Crassignatha* species were borrowed from the Life College of Sciences, Hunan Normal University in Changsha (HNU), China and the Institute of Zoology, Chinese Academy of Sciences in Beijing (IZCAS), China. All materials were examined using a Leica M205 C stereomicroscope and photographed with a Canon EOS 60D wide zoom digital camera (8.5 megapixels) mounted on an Olympus BX 43 compound microscope. Male palps and epigynes were examined and photographed after dissection. The left palp was photographed and described unless it is missing, then the right was selected. Epigynes were treated with lactic acid before being embedded in Hoyer’s gum to take photos of the vulva. The images were montaged using Helicon Focus 3.10 (Khmelik et al. 2006) image stacking software. All measurements are in millimeters. Leg measurements are given as follows: total length (femur, patella, tibia, metatarsus, and tarsus).

Tissue samples were taken from twenty-eight individuals of *Crassignatha*, including eleven new and six known species. Molecular data were obtained from specimens collected at the type locality, although not from the type specimens themselves. A partial fragment (636 bp) of the mitochondrial gene cytochrome c oxidase subunit I (COI) was amplified and sequenced to calculate the genetic distances between morphologically similar species and to confirm identifications and sex pairing accuracy.

The primers used are: LCO1490 (5'-GGTCAACAAATCATCATAAAGATATTGG-3') and HCO2198 (5'-TAAACTTCAGGGTGACCAAAAAATCA-3'). Raw sequences were edited and assembled using BioEdit v.7.2.5 (Hall 1999), and the uncorrected pairwise distances between species were calculated using MEGA7.0.14 (Kumar et al. 2016). Results of the genetic distance analysis are shown in Appendix 1.

Abbreviations used in the text or figures are given in Table 1. References to figures in the cited papers are in lowercase (fig. or figs), figures in this paper are noted with an initial capital (Fig. or Figs). New sequences generated for this study are available in GenBank, and the accession numbers are reported in Table 2. With the exception of the types of previously described species kept in **HNU** and **IZCAS**, all molecular vouchers are tentatively deposited in **NHMSU** in Chengdu, China, and examined morphological material is deposited in **NHMSU** and **IZCAS**.

**Table 1.** List of abbreviations used in the text or figures.

	Male palp		Epigyne
**C**	conductor	**CD**	copulatory duct
**CB**	cymbium	**CO**	copulatory opening
**CT**	cymbial tooth	**FD**	fertilization ducts
**E**	embolus	**S**	spermathecae
**EM**	embolic membrane	**Sp**	scape
**Fe**	femur		**Ocular area**
**MA**	median apophysis	**ALE**	anterior lateral eye
**Pa**	patella	**PLE**	posterior lateral eye
**T**	tegulum	**PME**	posterior median eye
**Ti**	tibia	**PER**	posterior eye row
**TS**	tibial spur on leg II		
	**Institutions**		
**HNU**	College of Life Sciences, Hunan Normal University, Changsha, China		
**IZCAS**	Institute of Zoology, Chinese Academy of Sciences, Beijing, China		
**NHMSU**	Natural History Museum of Sichuan University, Chengdu, China		

**Table 2.** GenBank accession numbers for new DNA sequence data from seventeen *Crassignatha* species.

Species	Identifier	Sex/Stage	COI	Collection localities
*Crassignatha baihua* sp. nov.	HA109	♂/adult	MT992007	China, Yunnan, Longling Co., Baihualing Village
	HA109	♀/adult	MT992006	
*Crassignatha bangbie* sp. nov.	HA137	♀/adult	MT992015	China, Yunnan, Longling Co., Bangbie Village
*Crassignatha dongnai* sp. nov.	HA092	♀/adult	MT992004	Vietnam, Dong Nai Pro., Cat Tien National Park
*Crassignatha ertou*	HA108	♀/adult	MT992005	China, Yunnan, Baoshan, Mangkuan Town, Baihualing
*Crassignatha gucheng* sp. nov.	HA132	♀/adult	MT992014	China, Yunnan, Longling Co., Mt. Xiaohei Nature Reserve
*Crassignatha mengla* sp. nov.	HA080	♂/juvenile	MT992000	China, Yunnan, Mengla Co., Baca Nature Reserve
	HA080	♀/adult	MT991999	
*Crassignatha nantou* sp. nov.	HA055	♂/adult	MT991996	China, Taiwan, Nantou Co., Hehuan Hill
	HA055	♀/adult	MT991995	
*Crassignatha nasalis* sp. nov.	HA041	♂/adult	MT991992	China, Sichuan, Guling Co., Yuhua Town, Taoyuan Cave
	HA041	♀/adult	MT991991	
*Crassignatha pianma*	HA113	♂/adult	MT992009	China, Yunnan, Lushui Co., Pianma Town, broad-leaf forest
	HA113	♀/adult	MT992008	
*Crassignatha quadriventris*	HA025	♀/adult	MT991990	China, Hainan, Dongfang City, Nanlang Village, E’xianling
*Crassignatha rostriformis* sp. nov.	HA079	♂/adult	MT991998	China, Yunnan, Xichou Co., Xianren Cave
	HA079	♀/adult	MT991997	
*Crassignatha shiluensis*	HA081	♂/juvenile	MT992002	China, Yunnan, Mengla Co., Xishuangbanna Botanical Garden
	HA081	♀/adult	MT992001	
*Crassignatha shunani* sp. nov.	HA046	♂/juvenile	MT991994	China, Sichuan, Guling Co., Dahei Cave
	HA046	♀/adult	MT991993	
*Crassignatha si* sp. nov.	HA141	♂/juvenile	MT992017	China, Yunnan, Yiliang Co., Dazhezong Village, Baiyan Cave
	HA141	♀/juvenile	MT992016	
*Crassignatha thamphra* sp. nov.	HA089	♀/adult	MT992003	Thailand, Khon Kaen Pro., Phu Pha Man Distr., Tham Phra Cave
*Crassignatha yamu*	HA115	♂/adult	MT992011	China, Yunnan, Fugong Co., Shilajia Village, Yamu River
	HA115	♀/adult	MT992010	
*Crassignatha yinzhi*	HA117	♂/adult	MT992013	China, Yunnan, Longling Co., Mt. Xiaohei Nature Reserve
	HA117	♀/adult	MT992012	

## Taxonomy

### Family Symphytognathidae Hickman, 1931

#### Genus Crassignatha

Taxon classificationAnimaliaAraneaeSymphytognathidae

Wunderlich, 1995

8AC2AD48-4E59-5962-A251-AE921F6DAD1D

Crassignatha Wunderlich, 1995: 546
Crassignatha Miller et al., 2009: 68

##### Type species.

*Crassignatha haeneli* Wunderlich, 1995 by original designation, from Malaysia.

##### Diagnosis.

**Figure 1.**
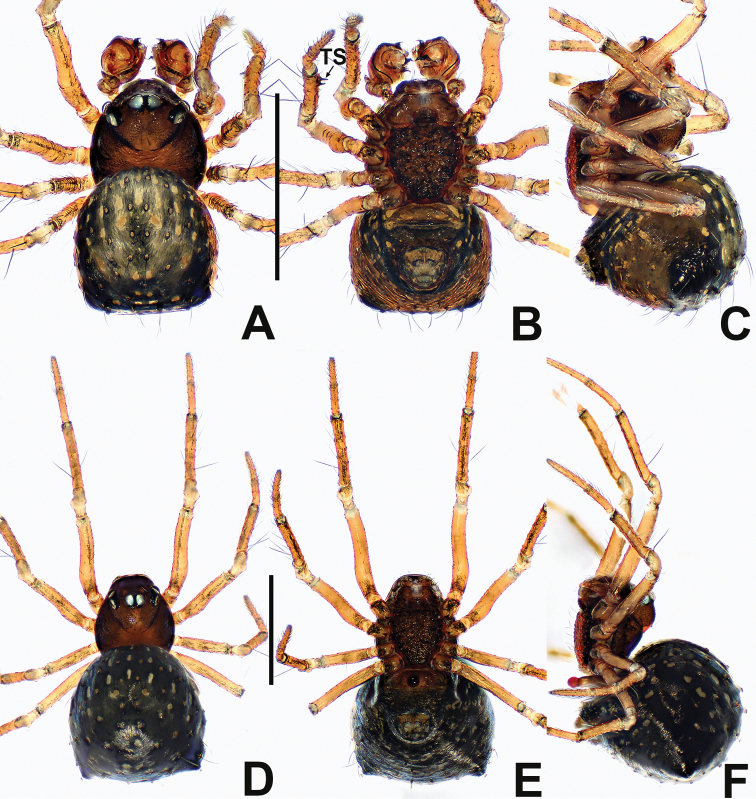
*Crassignatha baihua* sp. nov. **A** male habitus, dorsal **B** male habitus, ventral **C** male habitus, lateral **D** female habitus, dorsal **E** female habitus, ventral **F** female habitus, lateral. Scale bars: 0.50 mm (**A–F**).

*Crassignatha* can be distinguished from *Anapistula* Gertsch, 1941 by having six eyes vs. four or absent in the latter; and from *Anapogonia* Simon, 1905 by the chelicerae fused near the base vs. unfused. (The latter is tentatively placed in Symphytognathidae (Plantnick and Forster 1989: 76)). *Crassignatha* differs from *Globignatha* Balogh & Loksa, 1968 and *Symphytognatha* Hickman, 1931 by the chelicerae fused only near the base vs. almost fully fused in the latter two (Balogh and Loksa 1968: fig. 10; Forster and Platnick 1977: fig. 41; Lin 2019: fig. 1H). *Crassignatha* is most similar to *Curimagua* Forster & Platnick, 1977 and *Patu* Marples, 1951 in habitus features and body size but differs from *Curimagua* by having six eyes in diads and lacking female palps rather than eyes in triads and female palps reduced to remnants but not absent (Fig. 1A, D vs. Forster and Platnick 1977: figs 40, 63); and from *Patu* by the sculptured carapace (Fig. 16A, D; Wunderlich 1995: fig. 15; smooth in a few species) and the male abdomen usually with a lateral scutum (Fig. 1C; absent in a few species).

##### Description.

Body length 0.50–0.90 in male, 0.60–1.30 in female; six eyes in three diads. Ocular area in male raised more than in female. Carapace sub-rounded or pear shaped, brown or yellow-brown, usually sculptured on surface, but smooth in a few species. Cervical groove distinct. Clypeus concave. Chelicerae usually fused near base, with one or two retromarginal teeth. Labium triangular or semilunar, fused to sternum. Sternum scutellate or heart shaped, slightly plump, surface mostly sculptured, rarely smooth, truncated posteriorly. Legs pale yellow to brown-yellow. Leg formula: I-II-IV-III or I-VI-II-III. Male tibia II usually with two long clasping spurs on ventral-subdistal part (but only one spur in a few species). Abdomen globular or quadrate posteriorly in both sexes, male usually with weakly sclerotized abdominal scutum laterally and posteriorly (absent in few species), with an annular plate around spinnerets. Colulus absent.

**Male** palps oblate. Cymbium wraps around bulb on the prolateral-ventral surface, with a distal cymbial tooth. Median apophysis present, conductor absent. Embolus sclerotized, usually attached to a transparent embolic membrane at base.

**Female** genital area weakly sclerotized, internal structure faintly visible through tegument. Majority of species with protruded scape, copulatory opening located at apex of scape. Paired spermathecae globular, separated. Copulatory ducts tortile, usually connected to the posterolateral or dorsal surface of spermathecae. Fertilization ducts usually starting at the posterior or lower inner surface of spermathecae.

##### Composition.

*Crassignatha baihua* sp. nov., *C. bangbie* sp. nov., *C. bicorniventris* (Lin & Li, 2009), *C. changyan* sp. nov., *C. danaugirangensis*Miller et al., 2014, *C. dongnai* sp. nov., *C. ertou* Miller, Griswold & Yin, 2009, *C. gucheng* sp. nov., *C. gudu* Miller, Griswold & Yin, 2009, *C. haeneli* Wunderlich, 1995, *C. mengla* sp. nov., *C. nantou* sp. nov., *C. nasalis* sp. nov., *C. pianma* Miller, Griswold & Yin, 2009, *C. quadriventris* (Lin & Li, 2009), *C. quanqu* Miller, Griswold & Yin, 2009, *C. rostriformis* sp. nov., *C. shiluensis* (Lin & Li, 2009), *C. shunani* sp. nov., *C. si* sp. nov., *C. thamphra* sp. nov., *C. xichou* sp. nov., *C. yamu* Miller, Griswold & Yin, 2009, and *C. yinzhi* Miller, Griswold & Yin, 2009. *Patu bispina* Lin, Pham & Li, 2009, and *P. kishidai* Shinkai, 2009 may also belong in this genus.

##### Distribution.

Southern China (Guizhou, Yunnan, Hainan, and Taiwan), Central Japan (Honshu, Shikoku), Vietnam, Thailand, Malaysia.

#### Key to species of Crassignatha Wunderlich, 1995

**Table:** 

1	Males	**2**
–	Females	**18**
2	Embolus long, extending beyond anterior edge of median apophysis (Figs 8B, 15B, 27A, 31B, 35B)	**3**
–	Embolus short, not extending beyond anterior edge of median apophysis (Figs 10B, 21B, 23B)	**13**
3	Embolus filiform, flexible (Figs 27A, 35B, 37A, B)	**4**
–	Embolus spiraled, stiff (Figs 8B, 15B, 31B)	**8**
4	Embolus coiled (Figs 27A, 35B)	**5**
–	Embolus not coiled (Fig. 37A; Wunderlich 1995: fig. 19; Miller et al. 2014: fig. 4)	**6**
5	Embolus coiled into more than two loops (Fig. 27A)	***C. shiluensis***
–	Embolus coiled into fewer than two loops (Fig. 35B)	***C. yamu***
6	Embolus filiform, without basal nodule	**7**
–	Embolus straight, pointed, with a basal nodule (Fig. 37A, B)	***C. yinzhi***
7	Median apophysis bilobate (Wunderlich 1995: fig 18)	***C. haeneli***
–	Median apophysis trilobate (Miller et al. 2014: fig. 4)	***C. danaugirangensis***
8	Embolus twisted anticlockwise (Figs 8B, 15B, 25B)	**9**
–	Embolus twisted clockwise (Fig. 31B)	***C. si* sp. nov.**
9	Cymbial tooth large, hook shaped (Figs 8B, 25B)	**10**
–	Cymbial tooth small, tooth-like (Figs 15B, 17A, 29A)	**11**
10	Embolic base narrow (Fig. 8B)….	***C. ertou***
–	Embolic base wide (Fig. 25B)	***C. rostriformis* sp. nov.**
11	Embolic tip blunt, stiff (Fig. 17B)	***C. nasalis* sp. nov.**
–	Embolic tip sharp, narrow (Figs 15A, B, 29A, B)	**12**
12	Cymbial tooth sharp, median apophysis lacks a hook (Fig. 29A)	***C. shunani* sp. nov.**
–	Cymbial tooth blunt, median apophysis with a hook (Fig. 15A)	***C. nantou* sp. nov.**
13	Embolic tip blunt (Figs 10B, 19B, 23B)	**14**
–	Embolic tip pointed (Figs 2B, 13B, 21B)	**16**
14	Embolic base wide; cymbial tooth spur-like (Figs 19A, B, 23A, B)	**15**
–	Embolic base narrow; cymbial tooth spine-like (Fig. 10A, B)	***C. gucheng* sp. nov.**
15	Median apophysis subquadrate, with a hooked process (Fig. 19A, B)	***C. pianma***
–	Median apophysis subtriangular, with a truncated process (Fig. 23A, B)	***C. quanqu***
16	Embolic tip narrow; median apophysis with a process (Figs 2B, 21B)	**17**
–	Embolic tip wide; median apophysis lacks a process (Fig. 13A, B)	***C. mengla* sp. nov.**
17	Embolic apex flat (Fig. 2B)	***C. baihua* sp. nov.**
–	Embolic apex sloped (Fig. 21A, B)	***C. quadriventris***
18	Scape long, distinctly protrudes from epigastric furrow (Figs 2D, 6D, 10D)	**19**
–	Scape short or absent (Figs 4D, 5D, 27C, 33D)	**27**
19	Copulatory ducts merged into a tubular atrium medially (Figs 11F, 35G)	**20**
–	Copulatory duct junction near copulatory opening (Figs 2F, 8F, 10F)	**22**
20	Copulatory atrium short, does not extend beyond spermathecal anterior margin	**21**
–	Copulatory atrium long, extends beyond spermathecal anterior margin (Fig. 31F)	***C. si* sp. nov.**
21	Copulatory ducts do not overlap atrium (Fig. 11F, G)	***C. gudu***
–	Copulatory ducts overlap part of atrium (Fig. 35F, G)	***C. yamu***
22	Copulatory ducts form a V-shape before junction (Figs 8F, 37F)	**23**
–	Copulatory ducts nearly parallel at center before junction (Figs 2F, 21F)	**24**
23	Copulatory ducts make two sharp turns (Fig. 37F)	***C. yinzhi***
–	Copulatory ducts make four sharp turns (Fig. 8F)	***C. ertou***
24	Copulatory duct connects to spermathecal dorsum (Figs 6G, 10G)	**25**
–	Copulatory duct connects to posterior margin of spermathecae (Fig. 2G, 21G)	**26**
25	Copulatory ducts twisted twice in center of vulva (Fig. 10F, G)	***C. gucheng* sp. nov.**
–	Copulatory ducts twisted once in center of vulva (Fig. 6F, G)	***C. dongnai* sp. nov.**
26	Copulatory ducts make four turns (Fig. 2F, G)	***C. baihua* sp. nov.**
–	Copulatory ducts make six turns (Fig. 21F, G)	***C. quadriventris***
27	Scape absent (Figs 4D, 17D, 27C, 33D)	**28**
–	Scape present (Figs 3E, 19E, 29E, 32E, Miller et al. 2014: fig. 2)	**33**
28	Copulatory duct loops 3 ×, connects anterolaterally to spermathecae (Fig. 27D, E)	***C. shiluensis***
–	Vulva not as above	**29**
29	Spermathecae separated by at least their diameter (Figs 5E, 33E)	**30**
–	Spermathecae separated by less than their diameter (Figs 4F, 15F, 17E)	**31**
30	Copulatory ducts vertically linked to copulatory opening (Fig. 33E)	***C. xichou* sp. nov.**
–	Copulatory ducts diagonally linked to copulatory opening (Fig. 5E)	***C. changyan* sp. nov.**
31	Copulatory ducts vertical and parallel in center of vulva (Fig. 15F)	***C. nantou* sp. nov.**
–	Copulatory ducts not as above	**32**
32	Copulatory ducts twisted 2× (Fig. 17E, F)	***C. nasalis* sp. nov.**
–	Copulatory ducts twisted 4× (Fig. 4F, G)	***C. bicorniventris***
33	Spermathecae separated by less than 1.5× their diameter	**34**
–	Spermathecae separated by more than 3× their diameter (Miller et al. 2014: fig. 2)	***C. danaugirangensis***
34	Copulatory ducts nearly vertically linked to copulatory opening (Figs 13F, 29F)	**35**
–	Copulatory ducts diagonally or horizontally linked to copulatory opening	**37**
35	Proximal part of copulatory ducts not confluent (Fig. 29F)	***C. shunani* sp. nov.**
–	Copulatory ducts confluent before reaching copulatory opening (Figs 13F, 25F)	**36**
36	Spermathecae separated by their diameter (Fig. 25F)	***C. mengla* sp. nov.**
–	Spermathecae spacing does not exceed their diameter (Fig. 32G)	***C. rostriformis* sp. nov.**
37	Copulatory ducts horizontally linked to copulatory opening	**38**
–	Copulatory ducts diagonally linked to copulatory opening	**39**
38	Copulatory duct has two inflection points in middle of vulva (Fig. 19F)	***C. pianma***
–	Copulatory duct has one inflection point in middle of vulva (Fig. 3F)	***C. bangbie* sp. nov.**
39	Proximal copulatory ducts curved (Fig. 23D)	***C. quanqu***
–	Proximal copulatory ducts straight (Fig. 32F)	***C. thamphra* sp. nov.**

#### Crassignatha baihua

Taxon classificationAnimaliaAraneaeSymphytognathidae

Y. Lin & S. Li sp. nov.

ED9A0910-DAC2-5D45-A2A3-7C972B7C4BBD

http://zoobank.org/02AB09F5-3549-4519-BE47-09EA3EB29A52

Figs 1 , 2 , 38

##### Type material.

***Holotype*** ♂ (NHMSU Ar 001) and ***paratypes*** 3♂ 8♀ (NHMSU Ar 002–012), **China**: Yunnan Province, Longling County, Mangkuan Township, Zaotanghe at Baihua Ling (Flowers Ridge) Village, in leaf litter under undisturbed subtropical broadleaf forest (25.30450°N, 98.80059°E; 1635 m), 21.VIII.2018, Y. Lin et al. leg. 1♂ (NHMSU-HA109) and 1♀ (NHMSU-HA109) used for sequencing, GenBank: MT992007 and MT992006, same data as for preceding.

##### Other material examined.

1♂ 13♀ (NHMSU-HA110), same data as holotype; 1♂ 2♀ (NHMSU-HA104), **China**: Yunnan Province, Nujiang Prefecture, Fugong County, Shiyueliang Township (27.27012°N, 98.89803°E; 1647 m), 5.VII.2016, Y. Li leg.

##### Diagnosis.

**Figure 2.**
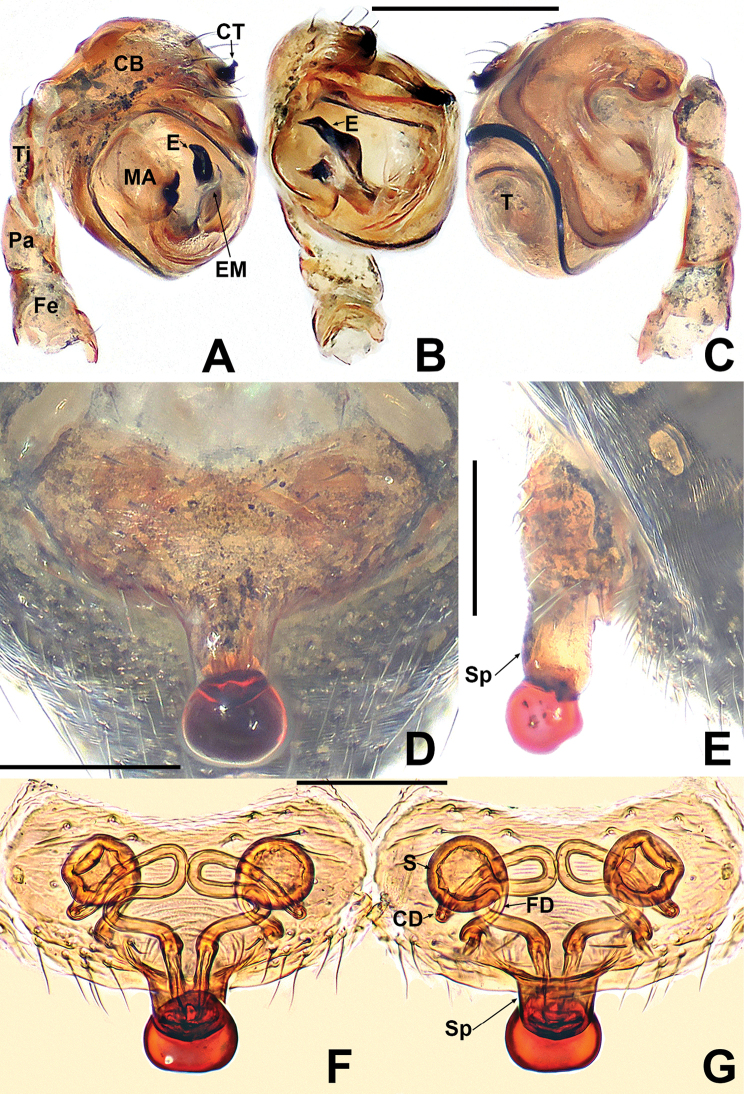
*Crassignatha baihua* sp. nov. **A** male palp, prolateral **B** male palp, ventral **C** male palp, retrolateral **D** epigyne, ventral **E** epigyne, lateral **F** vulva, ventral **G** vulva, dorsal. Scale bars: 0.10 mm (**A–G**).

*Crassignatha baihua* sp. nov. is similar to *C. quadriventris* but can be distinguished by the short, rigid, distally flat embolus (Fig. 2A) and the long copulatory ducts that make four turns before reaching the copulatory opening (Fig. 2F, G).

##### Description.

**Male** (holotype). Total length 0.64. Carapace 0.32 long, 0.32 wide, 0.36 high. Clypeus 0.10 high. Sternum 0.24 long, 0.20 wide. Abdomen 0.44 long, 0.44 wide, 0.48 high. Length of legs: I 0.94 (0.20, 0.10, 0.28, 0.16, 0.20); II 0.84 (0.16, 0.10, 0.24, 0.14, 0.20); III 0.60 (0.10, 0.06, 0.14, 0.12, 0.18); IV 0.68 (0.14, 0.06, 0.18, 0.10, 0.20).

**Somatic characters** (Fig. 1A–C). ***Coloration***: carapace, sternum, chelicerae, endites, and labium dark brown. Abdomen light black, with numerous small, sclerotized patches, with single orange scutum laterally and posteriorly. ***Prosoma***: carapace nearly pear shaped, surface granular, with two setae medially. Cephalic region elevated. PER strongly recurved. Chelicerae covered with setae anteriorly. Sternum almost heart shaped, rough, slightly swollen, truncated posteriorly, surface rough. ***Legs***: light brown, covered with setae and bristles. Tibia II with two clasping spurs. ***Abdomen***: anteriorly round, posteriorly square. Dorsally with pale yellow speckles, lateral scutum present, bears sparse, long setae. Spinnerets light brown, with circular plate.

***Palp*** (Fig. 2A–C): bulb relatively large, ~½ size of carapace. Cymbium bears apical setae, cymbial tooth hooked. Tegulum smooth, globular. Median apophysis nearly square, with a prolateral-distal process. Embolic membrane arises between median apophysis and embolus. Embolus short, rigid, distally blunt, forming a torsion.

**Female** (one of the paratypes). Total length 0.96. Carapace 0.36 long, 0.36 wide, 0.36 high. Clypeus 0.10 high. Sternum 0.24 long, 0.24 wide. Abdomen 0.64 long, 0.64 wide, 0.72 high. Length of legs: I 1.30 (0.42, 0.14, 0.30, 0.20, 0.24); II 1.06 (0.30, 0.14, 0.24, 0.16, 0.22); III 0.70 (0.18, 0.08, 0.20, 0.08, 0.16); IV 1.02 (0.30, 0.14, 0.24, 0.14, 0.20).

**Somatic characters** (Fig. 1D–F). ***Coloration***: carapace, sternum, chelicerae, endites, and labium dark brown. Abdomen black, with numerous small sclerotized patches. ***Prosoma***: carapace nearly pear shaped with small tubercles, with two strong setae medially. Cephalic region elevated. PER straight. Mouthparts and sternum as in male. ***Abdomen***: anteriorly rounded, posteriorly square, with paired posterolateral small tubercles. Spinnerets as in male, circular plate absent.

***Epigyne*** (Fig. 2D–G): epigynal area weakly sclerotized, with some setae. Scape distinctly extends beyond the epigastric furrow. Internal structures more or less visible via translucent tegument. Spermathecae separated by 1.2× their diameter. Fertilization ducts starting at posterior of spermathecae. Copulatory ducts long, connected to dorsal surface of spermathecae, forming four turns before curving downward. Subproximal copulatory ducts parallel, throughout the entire scape. Copulatory opening located at end of scape.

##### Etymology.

The specific epithet is derived from the type locality; noun in apposition.

##### Distribution.

China (Yunnan) (Fig. 38).

#### Crassignatha bangbie

Taxon classificationAnimaliaAraneaeSymphytognathidae

Y. Lin & S. Li sp. nov.

E7FCE439-B091-53B8-8DE7-283D84A4B207

http://zoobank.org/6E8AD84E-DD0B-437C-B5D2-FA67A28D67EB

Figs 3 , 38

##### Type material.

***Holotype*** ♀ (NHMSU Ar 013), **China**: Yunnan Province, Longling County, Zhen’an Township, Bangbie Village, at stream at km 6.8 on Road S317, shady embankments along stream, dusting webs in understory (24.81333°N, 98.83280°E; 1560 m), 22.VIII.2018, Y. Lin et al. leg.; 1♀ (NHMSU-HA137) used for sequencing, GenBank: MT992015, same data as for preceding.

##### Diagnosis.

**Figure 3.**
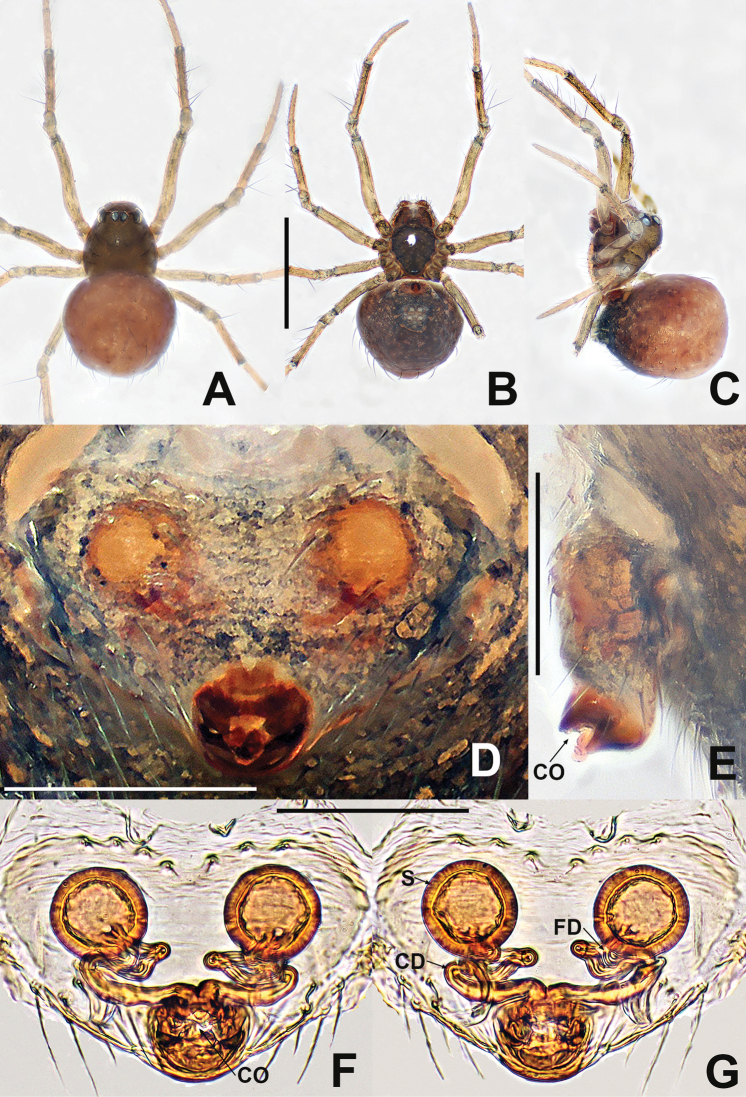
Female of *Crassignatha bangbie* sp. nov. **A** habitus, dorsal **B** habitus, ventral **C** habitus, lateral **D** epigyne, ventral **E** epigyne, lateral **F** vulva, ventral **G** vulva, dorsal. Scale bars: 0.50 mm (**A–C**); 0.10 mm (**D–G**).

*Crassignatha bangbie* sp. nov. is similar to *C. pianma* but can be distinguished by the copulatory duct having one inflection point in middle of vulva rather than two inflection points as in the latter (Fig. 3F, G).

##### Description.

**Female** (holotype). Total length 0.80. Carapace 0.32 long, 0.36 wide, 0.32 high. Clypeus 0.14 high. Sternum 0.20 long, 0.20 wide. Abdomen 0.52 long, 0.56 wide, 0.60 high. Length of legs: I 1.16 (0.40, 0.12, 0.28, 0.16, 0.20); II 0.92 (0.28, 0.12, 0.24, 0.12, 0.16); III 0.84 (0.24, 0.12, 0.16, 0.12, 0.20); IV 0.96 (0.32, 0.08, 0.24, 0.16, 0.16).

**Somatic characters** (Fig. 3A–C). ***Coloration***: carapace, sternum, chelicerae, endites, and labium brown. Abdomen dark orange with some light patches. ***Prosoma***: carapace nearly pear shaped. PER straight. Chelicerae covered with setae anteriorly. Labium nearly semicircular. Sternum smooth, bears sparse setae and small light patches, subcordate, truncated posteriorly. ***Legs***: brown with a little black, covered with setae and bristles. ***Abdomen***: anteriorly round, posteriorly relatively pointed, with light patches. Spinnerets gray, anterior spinnerets larger than posterior spinnerets.

***Epigyne*** (Fig. 3D–G): epigynal area slightly sclerotized, with few setae. Scape stubby, protruded. Internal structures faintly visible via translucent tegument. Spermathecae separated by approximately their diameter. Fertilization ducts starting at posterior margin of spermathecae. Copulatory ducts connected to the dorsal-subcentral surface of spermathecae, bent toward the central area of vulva to form an inflection point, then retracing under the spermathecae, fused at the copulatory opening. Copulatory openings large, rounded in ventral view, located at end of scape.

**Male.** Unknown.

##### Etymology.

The specific name is derived from the type locality; noun in apposition.

##### Distribution.

China (Yunnan) (Fig. 38).

#### Crassignatha bicorniventris

Taxon classificationAnimaliaAraneaeSymphytognathidae

(Lin & Li, 2009) comb. nov.

FCA5E7EC-D3B0-530B-A85C-D860F5AA9E14

Figs 4 , 38

Patu bicorniventris Lin & Li, 2009: 50, figs 1, 2A–D (♀).

##### Type material.

***Holotype*** ♀ and ***paratype*** 1♀ (IZCAS), **China**: Hainan Province, Changjiang Lizu Autonomous County, Qicha Town, Bawangling Nature Reserve (19.03333°N, 109.10000°E; 698 m), 29.VII.2007, S. Li and C. Wang leg. Examined.

##### Other material examined.

1♀ (IZCAS-Ar 40996), **China**: Hainan Province, Dongfang City, Donghe Town, Yalong Village, outside of Yalong Huangxian Cave (18.97920°N, 108.88967°E; 264 m), 15.XII.2014, Q. Zhao and L. Shao leg.

##### Diagnosis.

**Figure 4.**
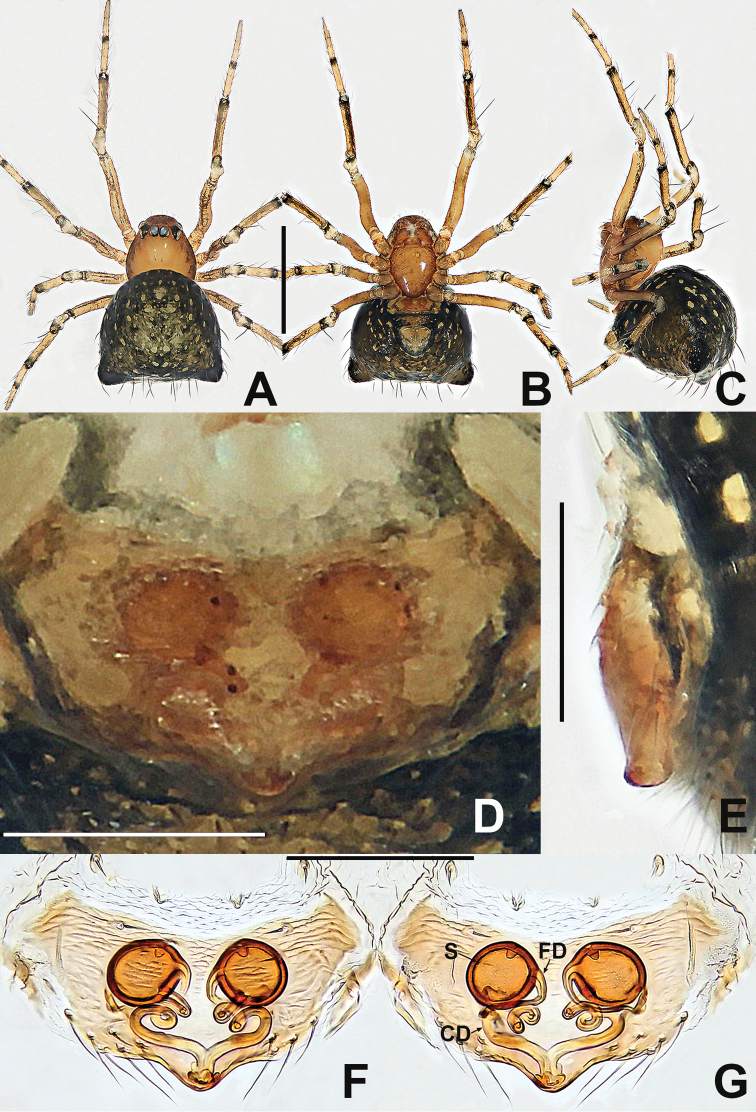
Female of *Crassignatha bicorniventris***A** habitus, dorsal **B** habitus, ventral **C** habitus, lateral **D** epigyne, ventral **E** epigyne, lateral **F** vulva, ventral **G** vulva, dorsal. Scale bars: 0.50 mm (**A–C**); 0.10 mm (**D–G**).

This species resembles *C. nasalis* sp. nov. but can be distinguished by the copulatory ducts twisting 4× but only 2× in the latter (Fig. 4D–G).

##### Description.

**Female** (IZCAS-Ar 40996). Total length 0.80. Carapace 0.36 long, 0.32 wide, 0.32 high. Clypeus 0.12 high. Sternum 0.28 long, 0.20 wide. Abdomen 0.56 long, 0.60 wide, 0.56 high. Length of legs: I 1.16 (0.40, 0.14, 0.26, 0.18, 0.18); II 1.00 (0.32, 0.10, 0.24, 0.14, 0.20); III 0.68 (0.20, 0.10, 0.12, 0.12, 0.14); IV 0.98 (0.34, 0.12, 0.20, 0.14, 0.18).

**Somatic characters** (Fig. 4A–C). ***Coloration***: carapace light brown, centrally smooth, marginally darkish, without modified pattern. Chelicerae, endites, labium, and sternum light brown. Abdomen charcoal gray, with irregular light patches. ***Prosoma***: carapace nearly pear shaped, with two strong setae medially. Cephalic region elevated. PER straight. Chelicerae covered with setae anteriorly. Sternum smooth, slightly plump, truncated posteriorly, fused to labium. ***Legs***: light brown, each segment with distal black ring, covered with setae and bristles. ***Abdomen***: anteriorly rounded, posteriorly quadrate, bears sparse, long setae, with a pair of posterolateral abdominal tubercles. Spinnerets light brown.

***Epigyne*** (Fig. 4D–G): epigynal area lightly sclerotized, with some setae. Internal structures faintly visible via translucent tegument. Scape small, unobvious. Paired spermathecae separated by ~ ½ their diameter. Fertilization ducts originating inside middle margin of spermathecae, bent downward, reverse course under the spermathecae. Copulatory opening small, located at end of scape. Copulatory ducts long, connected to posteriorly ventral surface of spermathecae, twisted 4× under spermathecae. Bilateral copulatory ducts merged into a Y-shaped atrium before copulatory opening.

**Male.** Unknown.

##### Taxonomic justification.

Although we were unable to obtain molecular data for this species, the configuration of the vulva and the modified habitus leave little doubt that it is a member of the genus *Crassignatha* and not *Patu*. Therefore, we propose a new combination, *Crassignatha bicorniventris* (Lin & Li, 2009) comb. nov., transferring it from *Patu*.

##### Distribution.

China (Hainan) (Fig. 38).

#### Crassignatha changyan

Taxon classificationAnimaliaAraneaeSymphytognathidae

Y. Lin & S. Li sp. nov.

ADBE85E0-3990-5BF5-B290-C4D81B529E23

http://zoobank.org/A38088C0-07EC-487E-A343-A5553A682FB8

Figs 5 , 38

##### Type material.

***Holotype*** ♀ (NHMSU Ar 014) and ***paratypes*** 3♀ (NHMSU Ar 015–017), **China**: Yunnan Province, Lushui County, Pianma Township, Changyan River, 9.3 km ESE Pianma, mixed broadleaf deciduous and evergreen forest, dusting small webs near ground in forest understory (25.99363°N, 98.66651°E; 2470 m), 10.VIII.2018, Y. Lin et al. leg.

##### Diagnosis.

**Figure 5.**
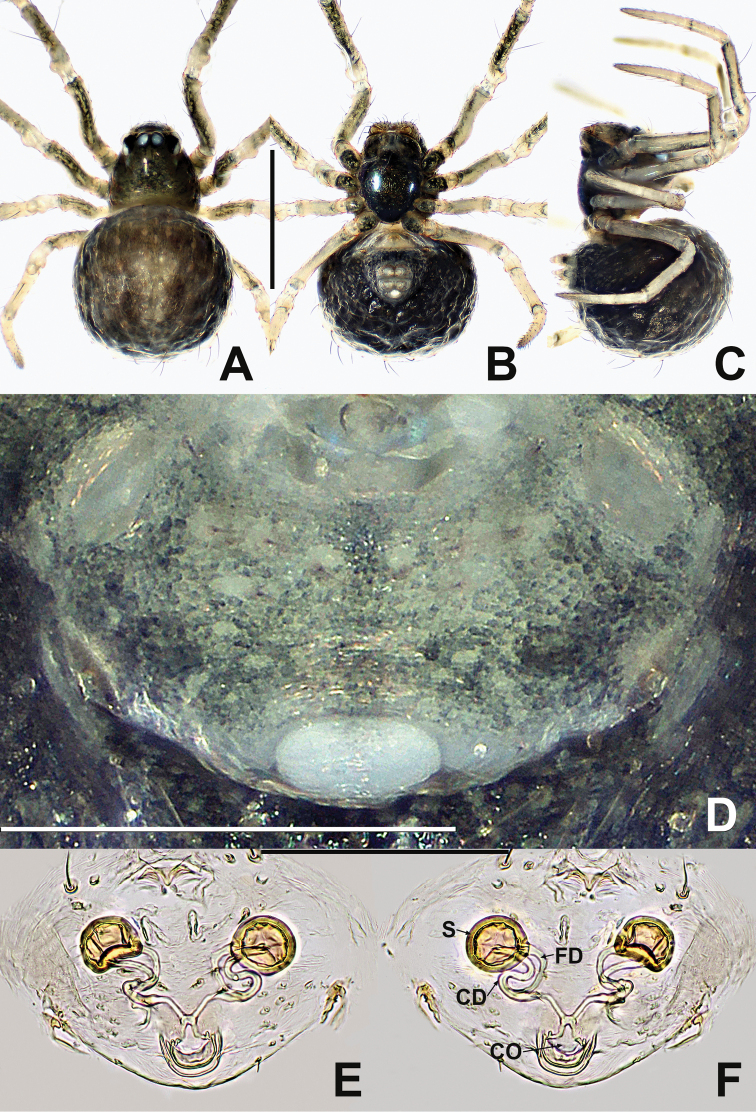
Female of *Crassignatha changyan* sp. nov. **A** habitus, dorsal **B** habitus, ventral **C** habitus, lateral **D** epigyne, ventral **E** vulva, ventral **F** vulva dorsal. Scale bars: 0.50 mm (**A–C**); 0.10 mm (**D–F**).

*Crassignatha changyan* sp. nov. is similar to *C. xichou* sp. nov. but differs by the copulatory ducts diagonally linked to the copulatory opening but vertically linked in the latter (Fig. 5E, F).

##### Description.

**Female** (holotype). Total length 0.88. Carapace 0.36 long, 0.32 wide, 0.36 high. Clypeus 0.14 high. Sternum 0.20 long, 0.20 wide. Abdomen 0.56 long, 0.56 wide, 0.60 high. Length of legs: I 1.12 (0.34, 0.20, 0.20, 0.14, 0.24); II 0.96 (0.32, 0.12, 0.16, 0.12, 0.24); III 0.78 (0.26, 0.10, 0.14, 0.08, 0.20); IV 0.98 (0.32, 0.12, 0.18, 0.12, 0.24).

**Somatic characters** (Fig. 5A–C). ***Coloration***: prosoma and abdomen dark, genital area and spinnerets slightly pale. Legs pale brown, with dark pigmentation. ***Prosoma***: carapace nearly pear shaped. PER recurved. Chelicerae bears setae anteriorly. Labium semilunar. Sternum heart shaped, smooth, slightly swollen, truncated posteriorly. ***Legs***: a distal seta on patella dorsally, two on tibia dorsally. ***Abdomen***: globose, modified by sparse, long setae and faint dots. Spinnerets distally pale.

***Epigyne*** (Fig. 5D–F): epigynal area with a few setae. Scape knobbed. Paired spermathecae globose, widely separated by at least 1.5× their diameter. Copulatory ducts connected to ventral center of spermathecae, twisting into an S-shaped under spermathecae, and merging anteriorly with copulatory openings. Copulatory openings located below scape. Fertilization ducts starting at inside margin of spermathecae, then curving posteriorly.

**Male.** Unknown.

##### Etymology.

The specific name is derived from the type locality; noun in apposition.

##### Distribution.

China (Yunnan) (Fig. 38).

#### Crassignatha dongnai

Taxon classificationAnimaliaAraneaeSymphytognathidae

Y. Lin & S. Li sp. nov.

9F826FAE-F002-5948-9845-038F7ADEDEDD

http://zoobank.org/AF9259E6-0DED-4890-BF12-804609E4F899

Figs 6 , 38

##### Type material.

***Holotype*** ♀ (IZCAS-Ar 40997) and ***paratype*** 1♀ (IZCAS-Ar 40998), **Vietnam**: Dong Nai Province, Cat Tien National Park, Natural Forest (11.45008°N, 107.36438°E; 173 m), 5.IX.2015, Q. Zhao, Y. Li and Z. Chen leg.; 1♀ (NHMSU-HA092) used for sequencing, GenBank: MT992004, same data as for preceding.

##### Diagnosis.

**Figure 6.**
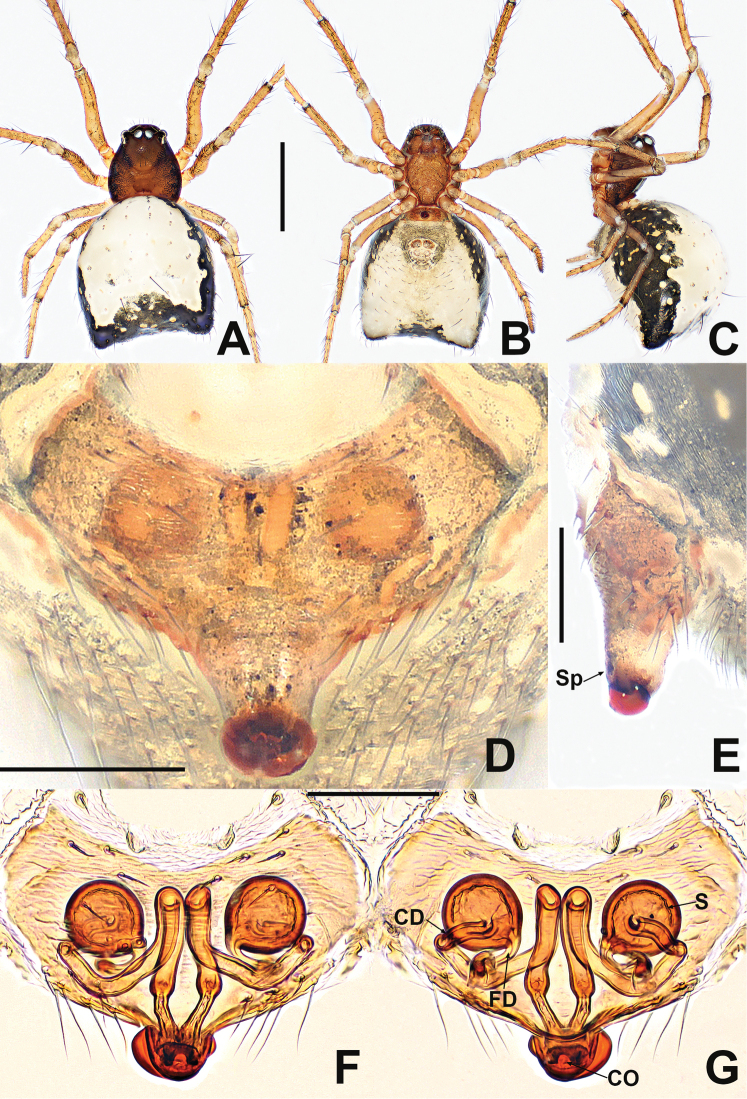
Female of *Crassignatha dongnai* sp. nov. **A** habitus, dorsal **B** habitus, ventral **C** habitus, lateral **D** epigyne, ventral **E** epigyne, lateral **F** vulva, ventral **G** vulva, dorsal. Scale bars: 0.50 mm (**A–C**); 0.10 mm (**D–G**).

This species is similar to *C. gucheng* sp. nov. but can be distinguished by the copulatory ducts twisted once in the center of vulva vs. twisted twice in the latter (Fig. 6F, G).

##### Description.

**Female** (holotype). Total length 1.28. Carapace 0.48 long, 0.40 wide, 0.40 high. Clypeus 0.14 high. Sternum 0.28 long, 0.28 wide. Abdomen 0.84 long, 0.76 wide, 0.80 high. Length of legs: I 1.60 (0.54, 0.18, 0.40, 0.24, 0.24); II 1.30 (0.40, 0.14, 0.32, 0.20, 0.24); III 1.00 (0.26, 0.14, 0.20, 0.16, 0.24); IV 1.20 (0.38, 0.14, 0.28, 0.18, 0.22).

**Somatic characters** (Fig. 6A–C). ***Coloration***: carapace brown, marginally darker, mouthparts and sternum brown. Legs pale brown. Abdomen pale dorsally and ventrally, black laterally and posteriorly. ***Prosoma***: carapace nearly pear shaped, granular with sulci, with two midline setae on cephalic area. Cephalic area elevated. PER recurved. Clypeus slightly concave. Labium tongue shaped, unfused to sternum. Sternum scutiform, flat, sculptured, truncated posteriorly. ***Legs***: patellae with dorsal seta distally, tibia with two dorsal setae. ***Abdomen***: anteriorly rounded, posteriorly subquadrate, with a pair of posterodorsal tubercles. Spinnerets slightly sclerotized.

***Epigyne*** (Fig. 6D–G): epigynal area distinctly sclerotized, bears a few setae. Scape large, finger-like, extended beyond the epigastric furrow. Internal structures faintly visible via the translucent tegument. Spermathecae separated by their diameter. Copulatory ducts long, connected to the dorsal center of spermathecae, curving downwards and going up to the center of vulva, then turning back vertically downward, merged before copulatory opening. Copulatory opening located at scape end. Fertilization ducts starting at inside lower margin of spermathecae, then curved posteriorly and laterally.

**Male.** Unknown.

##### Etymology.

The specific name is derived from the type locality; noun in apposition.

##### Distribution.

Vietnam (Fig. 38).

#### Crassignatha ertou

Taxon classificationAnimaliaAraneaeSymphytognathidae

Miller, Griswold & Yin, 2009

E0EC47F4-BC15-5C42-9D31-AF5C685013FC

Figs 7 , 8 , 38

Crassignatha ertou Miller et al., 2009: 74, figs 86D–F, 88A, B, 89A, B (♂♀).

##### Type materi﻿al.

***Holotype*** ♂ (HNU-CASENT 9029324) and ***paratypes*** 3♀ (HNU-CASENT 9022397), **China**: Yunnan Province, Longling County, Mangkuan Township, Zaotanghe at Baihualing Village, undisturbed subtropical broadleaf forest, dusting webs in understory (25.30450°N, 98.80059°E; 1635 m), 2.VI.2005, C. Griswold leg. Examined.

**Figure 7.**
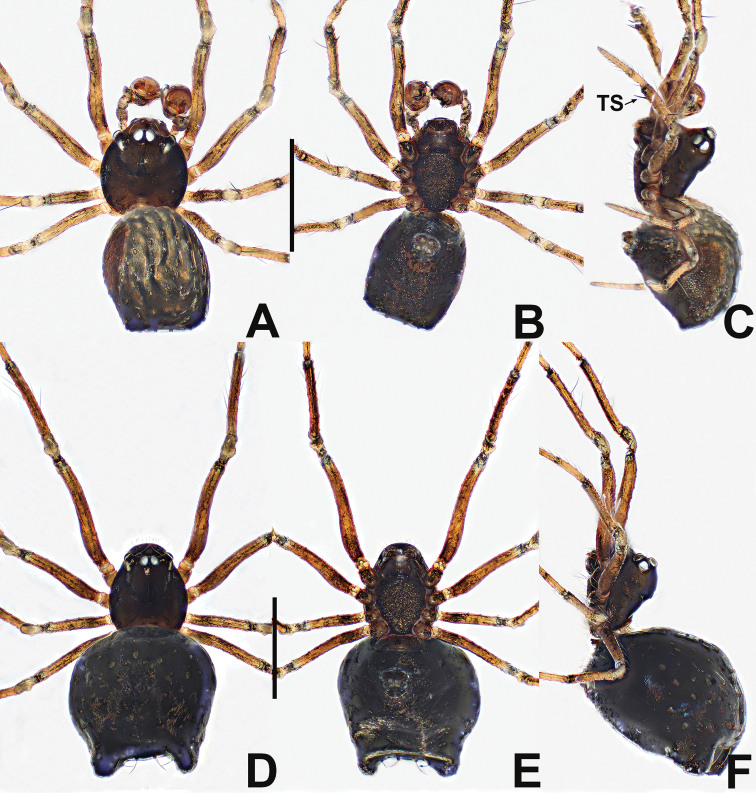
*Crassignatha ertou* Miller, Griswold & Yin, 2009 **A** male habitus, dorsal **B** male habitus, ventral **C** male habitus, lateral **D** female habitus, dorsal **E** female habitus, ventral **F** female habitus, lateral. Scale bars: 0.50 mm (**A–F**).

##### Other material examined.

1♂ 7♀ (NHMSU-HA108), **China**: Yunnan Province, Baoshan City, Longyang District, Mangkuan Township, Zaotanghe at Baihualing Village, good subtropical broadleaf forest, in leaf litter (25.30118°N, 98.79397°E; 1802 m), 18.VI.2016, Y. Li leg.; 1♀ (NHMSU-HA108) used for sequencing, GenBank: MT992005, same data as for preceding.

##### Diagnosis.

**Figure 8.**
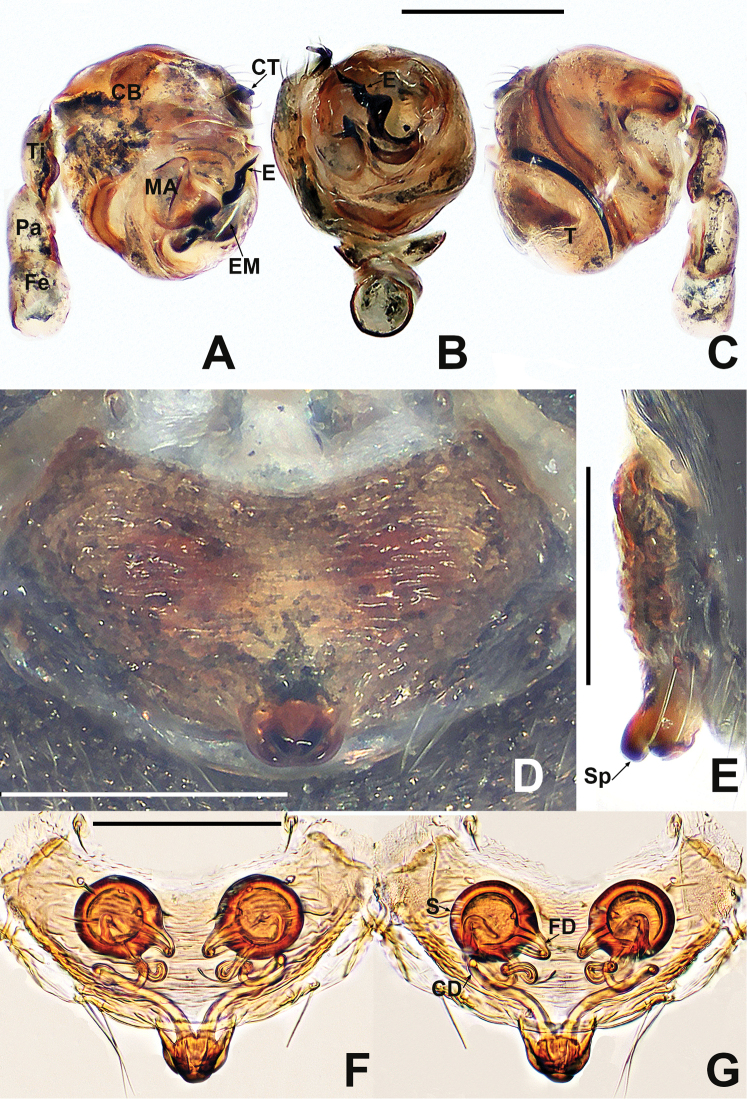
*Crassignatha ertou* Miller, Griswold & Yin, 2009 **A** male palp, prolateral **B** male palp, ventral **C** male palp, retrolateral **D** epigyne, ventral **E** epigyne, lateral **F** vulva, ventral **G** vulva, dorsal. Scale bars: 0.10 mm (**A–G**).

The male of *C. ertou* is similar to that of *C. rostriformis* sp. nov. but can be distinguished by the narrower embolic base, wider in the latter (Fig. 8A). The female of *C. ertou* is most similar to *C. yinzhi* but differs by the copulatory ducts having four sharp turns, but only two in the latter (Fig. 8D–G).

##### Description.

See Miller et al. (2009).

##### Distribution.

China (Yunnan) (Fig. 38).

#### Crassignatha gucheng

Taxon classificationAnimaliaAraneaeSymphytognathidae

Y. Lin & S. Li sp. nov.

C75BACDD-6F64-58F7-9DFD-C80D68C860DA

http://zoobank.org/0FF6F414-70C7-4404-9352-7AE564CA4971

Figs 9 , 10 , 38

##### Type material.

***Holotype*** ♂ (NHMSU Ar 018) and ***paratypes*** 2♂ 5♀ (NHMSU Ar 019–025), **China**: Yunnan Province, Longling County, Longjiang Township, Xiaoheishan Nature Reserve, Gucheng Mountain, in good forest (24.82886°N, 98.75917°E; 2010 m), 22.VIII.2018, Y. Lin et al. leg.; 1♀ (NHMSU-HA132) used for sequencing, GenBank: MT992014, same data as for preceding.

**Figure 9.**
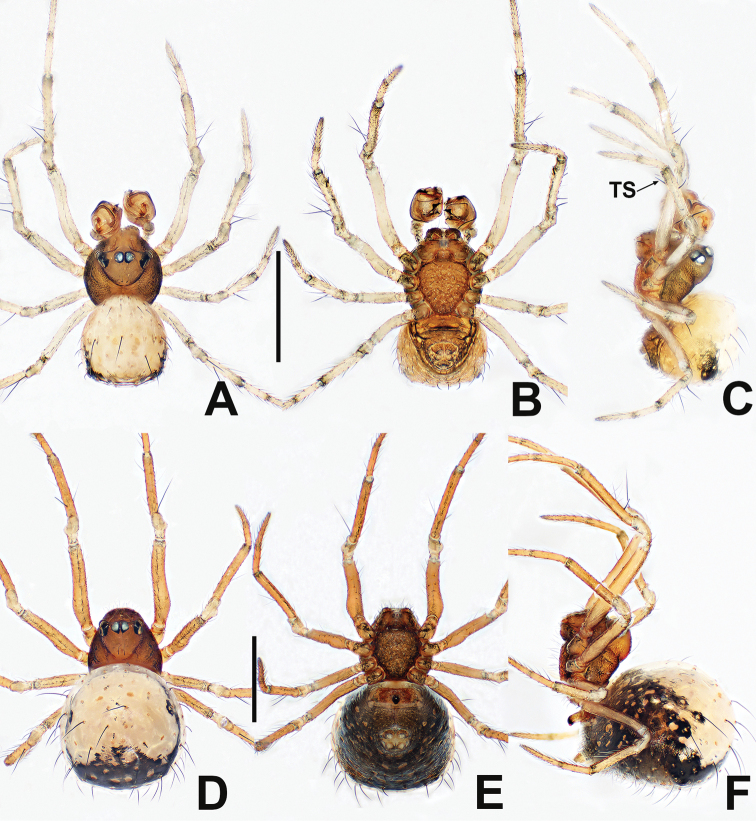
*Crassignatha gucheng* sp. nov. **A** male habitus, dorsal **B** male habitus, ventral **C** male habitus, lateral **D** female habitus, dorsal **E** female habitus, ventral **F** female habitus, lateral. Scale bars: 0.50 mm (**A–F**).

##### Diagnosis.

**Figure 10.**
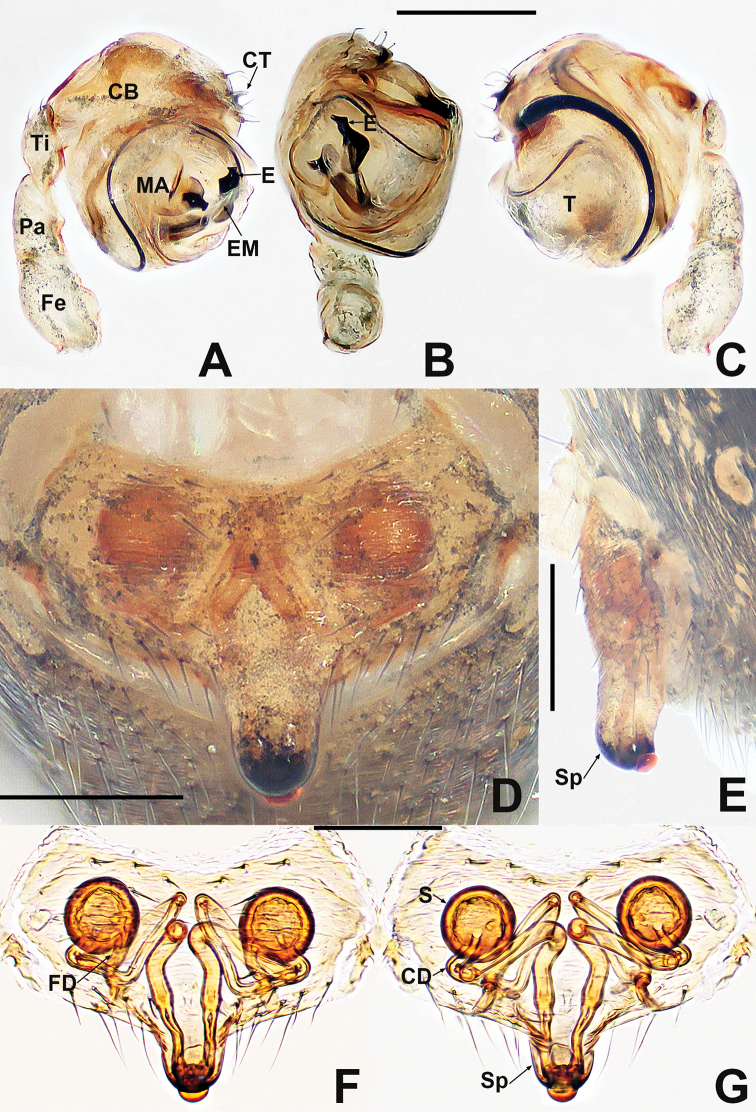
*Crassignatha gucheng* sp. nov. **A** male palp, prolateral **B** male palp, ventral **C** male palp, retrolateral **D** epigyne, ventral **E** epigyne, lateral **F** vulva, ventral **G** vulva, dorsal. Scale bars: 0.10 mm (**A–G**).

The male of *C. gucheng* sp. nov. is similar to that of *C. pianma* and *C. quanqu* but can be distinguished by the narrower embolic base and spine-like cymbial tooth vs. a wider embolic base and spur-like cymbial tooth in the latter two (Fig. 10A). The female of *C. gucheng* sp. nov. differs from that of *C. dongnai* sp. nov. by the copulatory ducts twisted twice at the center of vulva, only once in the latter (Fig. 10F, G).

##### Description.

**Male** (holotype). Total length 0.72. Carapace 0.32 long, 0.36 wide, 0.40 high. Clypeus 0.12 high. Sternum 0.24 long, 0.24 wide. Abdomen 0.44 long, 0.44 wide, 0.44 high. Length of legs: I 1.28 (0.38, 0.14, 0.32, 0.20, 0.24); II 0.98 (0.26, 0.14, 0.26, 0.14, 0.18); III 0.78 (0.20, 0.10, 0.16, 0.12, 0.20); IV 0.94 (0.30, 0.12, 0.18, 0.14, 0.20).

**Somatic characters** (Fig. 9A–C). ***Coloration***: carapace pale brown, marginally darker. Mouthparts and sternum pale brown. Legs pale yellow, with faint pigmentation on each segment distally. Abdomen pale dorsally, brown-yellow laterally and ventrally, dark posteriorly. ***Prosoma***: carapace nearly rounded, cephalic area elevated, cervical groove distinct, thoracic area and clypeus sculptured, clypeus concave. ALE protruded, PER recurved. Chelicerae bears setae anteriorly. Labium semicircular. Sternum heart shaped, slightly swollen, surface rough, truncated posteriorly. ***Legs***: with dorsal seta on each patella distally, with two setae on each tibia subproximally. Tibia II with two clasping spurs. ***Abdomen***: anteriorly rounded, posteriorly subquadrate, lateral scutum inconspicuous. Spinnerets slightly sclerotized, lack circular plate.

***Palp*** (Fig. 10A–C): relatively large, no less than 1/3 carapace size. A few setae on cymbium distally. Apical cymbial tooth caniniform. Tegulum smooth, broad, globular. Plate-like median apophysis with a distal sclerotized process. Embolic membrane slender, laminar, translucent, arises from base of embolus. Embolus short and rigid, terminus blunt.

**Female** (one of the paratypes). Total length 1.08. Carapace 0.44 long, 0.40 wide, 0.40 high. Clypeus 0.12 high. Sternum 0.28 long, 0.24 wide. Abdomen 0.72 long, 0.72 wide, 0.84 high. Length of legs: I 1.48 (0.56, 0.16, 0.40, 0.14, 0.22); II 1.20 (0.38, 0.16, 0.34, 0.12, 0.20); III 0.84 (0.28, 0.14, 0.18, 0.10, 0.14); IV 1.1 (0.38, 0.14, 0.28, 0.18, 0.12).

**Somatic characters** (Fig. 9D–F). ***Coloration***: carapace roughly the same as in male. Legs pale brown. Abdomen with black pigmentation laterally, posteriorly, and ventrally. ***Prosoma***: carapace pear shaped, sculptured, cephalic area slightly lower than in male. PER recurved. Mouthparts and sternum as in male. ***Abdomen***: anteriorly rounded and posteriorly subquadrate, surface with weakly sclerotized hairy patches. Spinnerets as in male.

***Epigyne*** (Fig. 10D–G): epigynal area distinctly sclerotized, bears a few setae. Scape large, finger-like, protruded, copulatory opening located at its terminus. Internal structures faintly visible via translucent tegument. Spermathecae globose, strongly sclerotized, widely separated by 1.5× their diameter. Fertilization duct originates at medial posterior margin of spermathecae, extends to posterior margin of epigyne. Copulatory ducts long, connected to dorsal surface of spermathecae, extends below spermathecae toward the vulval center, and forms two return paths, then turns downward to copulatory opening.

##### Etymology.

The specific name is derived from the type locality; noun in apposition.

##### Distribution.

China (Yunnan), Vietnam (Fig. 38).

#### Crassignatha gudu

Taxon classificationAnimaliaAraneaeSymphytognathidae

Miller, Griswold & Yin, 2009

C1EDD1F3-374C-5FBD-8214-D69C782D1556

Figs 11 , 38

Crassignatha gudu Miller et al., 2009: 75, fig. 89C, D (♀).

##### Type material.

***Holotype*** ♀ (HNU-CASENT 9029318), **China**: Yunnan Province, Longling County, Mangkuan Township, Zaotanghe at Baihualing Village, good subtropical broadleaf forest, dusting webs in understory (25.30450°N, 98.80059°E; 1635 m), 2.VI.2005, C. Griswold leg. Examined.

##### Diagnosis.

**Figure 11.**
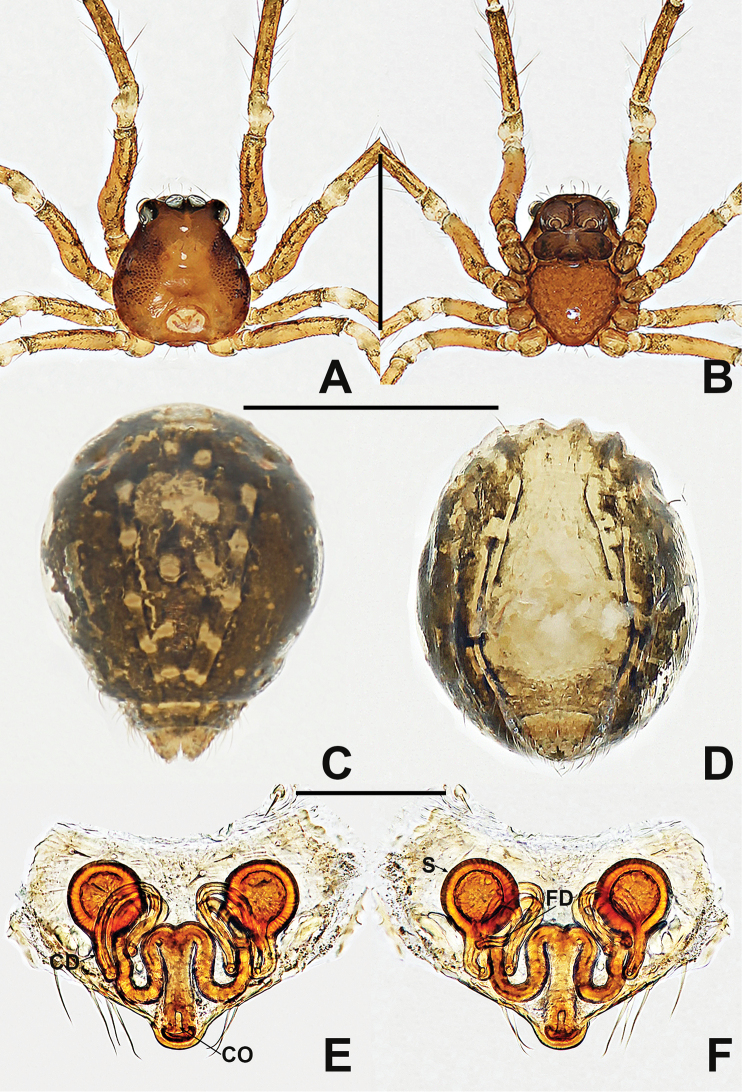
Female of *Crassignatha gudu***A** prosoma, dorsal **B** prosoma, ventral **C** abdomen, dorsal **D** abdomen, ventral **E** vulva, ventral **F** vulva, dorsal. Scale bars: 0.50 mm (**A–D**); 0.10 mm (**E, F**).

*Crassignatha gudu* can be easily distinguished from other congeners, except *C. yamu* and *C. si* sp. nov., by having a columnar atrium formed by the fusion of proximal copulatory ducts (Figs 11F, 31F, 35G). *Crassignatha gudu* is most similar to *C. yamu* and *C. si* sp. nov. and has a similar vulva configuration but differs by the longer copulatory atrium (Fig. 11E, F vs. Figs 31E, F, 35F, G).

##### Description.

See Miller et al. (2009).

##### Distribution.

China (Yunnan) (Fig. 38).

#### Crassignatha mengla

Taxon classificationAnimaliaAraneaeSymphytognathidae

Y. Lin & S. Li sp. nov.

7BB03011-D279-57CD-AE17-177283C24BAA

http://zoobank.org/8C586AFB-295C-4441-9C70-4E970A32BFD4

Figs 12 , 13 , 38

##### Type material.

***Holotype*** ♂ (IZCAS-Ar 40999) and ***paratypes*** 4♂ 8♀ (IZCAS-Ar 41000–41011), **China**: Yunnan Province, Xishuangbanna Prefecture, Mengla County, Menglun Town, Entrance to Shenmi Cave, in good forest (21.97332°N, 101.24336°E; 776 m), 3.X.2017, Y. Lin and Y. Li leg.; 1♂ juvenile (NHMSU-HA080) and 1♀ (NHMSU-HA080) used for sequencing, GenBank: MT992000 and MT991999, same data as for preceding.

##### Diagnosis.

The male of *Crassignatha mengla* sp. nov. is similar to that of *C. baihua* sp. nov. and *C. quadriventris* (Lin & Li, 2009) comb. nov. but differs by the wider and longer embolic tip and the median apophysis lacks a process, rather than a narrower (Fig. 13A, B), shorter embolic tip and a process on the median apophysis in the latter two (Figs 2A, B, 21A, B). The female of *C. mengla* sp. nov. is most similar to *C. rostriformis* sp. nov. in the vulva configuration but can be easily distinguished by the spermathecae separated by more than one diameter vs. less than one diameter (Figs 13F, G, 25E, F).

**Figure 12.**
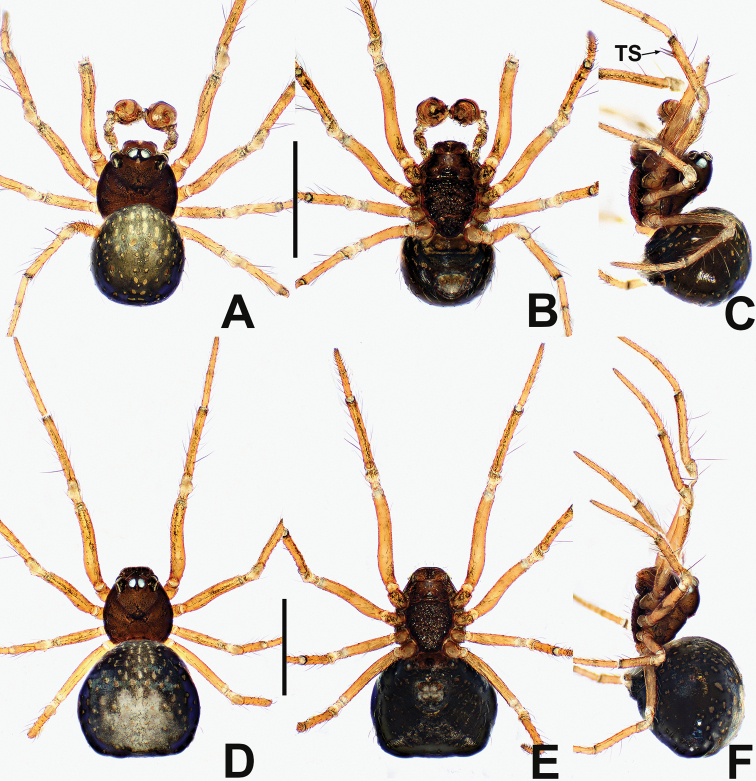
*Crassignatha mengla* sp. nov. **A** male habitus, dorsal **B** male habitus, ventral **C** male habitus, lateral **D** female habitus, dorsal **E** female habitus, ventral **F** female habitus, lateral. Scale bars: 0.50 mm (**A–F**).

##### Description.

**Male** (holotype). Total length 0.72. Carapace 0.36 long, 0.32 wide, 0.36 high. Clypeus 0.16 high. Sternum 0.24 long, 0.20 wide. Abdomen 0.44 long, 0.40 wide, 0.44 high. Length of legs: I 1.24 (0.38, 0.14, 0.30, 0.18, 0.24); II 1.04 (0.28, 0.14, 0.24, 0.14, 0.24); III 0.74 (0.18, 0.10, 0.14, 0.12, 0.20); IV 0.88 (0.24, 0.12, 0.20, 0.12, 0.20).

**Somatic characters** (Fig. 12A–C). ***Coloration***: prosoma dark brown. Legs brown-yellow. Abdomen charcoal gray, darker ventrally than dorsally. ***Prosoma***: carapace sub-rounded, surface rough, sculptured. Cephalic area elevated. ALE protruded, PER strongly recurved. Clypeus concave. Mouthparts strongly sclerotized. Labium semicircular. Sternum heart shaped, slightly plump, surface rough, truncated posteriorly. ***Legs***: tibia II with two clasping spurs subdistal-ventrally. ***Abdomen***: nearly rounded dorsally, with lightly sclerotized dots dorsally. Lateral scutum weakly sclerotized, dark brown. Spinnerets brown, with a circular plate.

**Figure 13.**
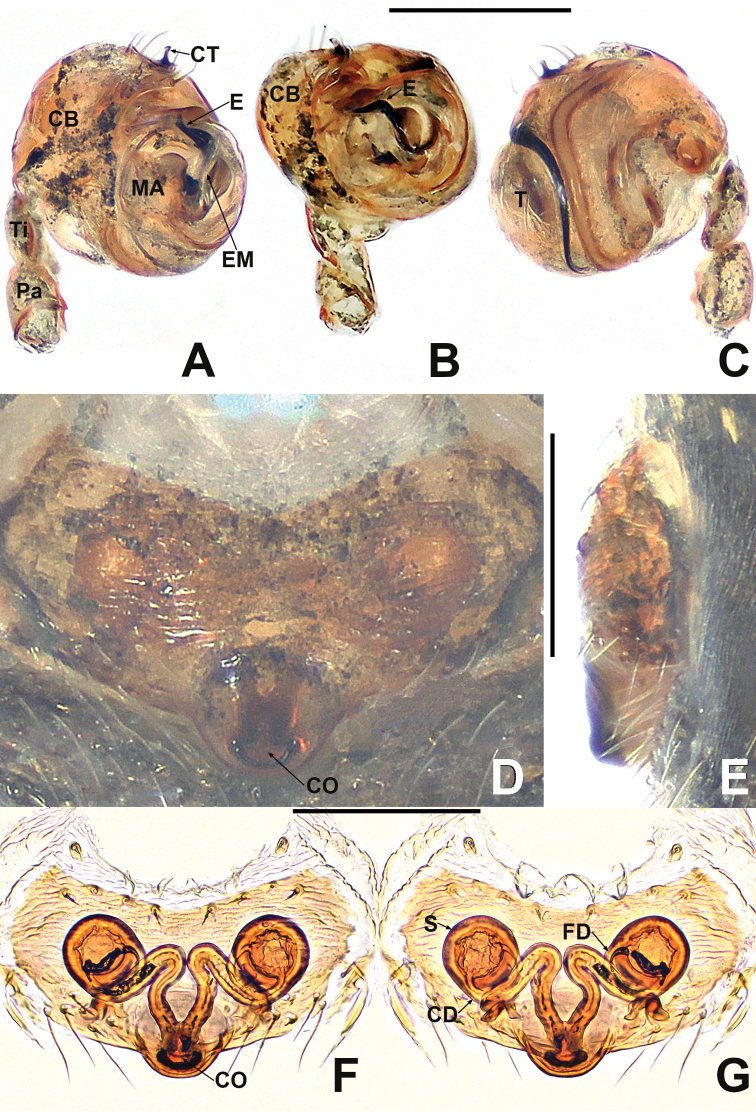
*Crassignatha mengla* sp. nov. **A** male palp, prolateral **B** male palp, ventral **C** male palp, retrolateral **D** epigyne, ventral **E** epigyne, lateral **F** vulva, ventral **G** vulva, dorsal. Scale bars: 0.10 mm (**A–G**).

***Palp*** (Fig. 13A–C): weakly sclerotized. Cymbium with few setae distally; cymbial tooth thin, located subapically. Tegulum globular and plump. Median apophysis lamellar, with sclerotized margin. Embolic membrane arises behind median apophysis, near embolic base. Embolus short, rigid, basolateral protrusion tapering distally, with a single bend.

**Female** (paratypes). Total length 1.00. Carapace 0.40 long, 0.36 wide, 0.36 high. Clypeus 0.14 high. Sternum 0.24 long, 0.24 wide. Abdomen 0.64 long, 0.64 wide, 0.60 high. Length of legs: I 1.38 (0.42, 0.14, 0.36, 0.20, 0.26); II 1.14 (0.30, 0.12, 0.30, 0.18, 0.24); III 0.88 (0.26, 0.10, 0.18, 0.14, 0.20); IV 1.10 (0.38, 0.12, 0.24, 0.14, 0.22).

**Somatic characters** (Fig. 12D–F). ***Coloration***: prosoma as in male. Abdomen lighter than in male. ***Prosoma***: carapace pear shaped. Cephalic region elevated, slightly lower than in male. PER slightly recurved. Labium triangular, unfused to sternum. Sternum heart shaped, surface rough, slightly plump, truncated posteriorly. ***Abdomen***: anteriorly round, posteriorly nearly square. Spinnerets brown, weakly sclerotized.

***Epigyne*** (Fig. 13D–G): epigynal area with few setae. Scape short, slightly protruded. Copulatory opening located at terminal part of scape. Internal structures faintly visible via translucent tegument. Spermathecae globose, widely separated by ~1.2× their diameter. Fertilization ducts short, thin, starting at the inside margin of spermathecae, deflexed, bifurcated distally. Copulatory ducts long, thick, connected to posterior margin of spermathecae, bent upward to center of vulva, then downward, fusing before reaching copulatory opening.

##### Etymology.

The specific name is derived from the type locality; noun in apposition.

##### Distribution.

China (Yunnan) (Fig. 38).

#### Crassignatha nantou

Taxon classificationAnimaliaAraneaeSymphytognathidae

Y. Lin & S. Li sp. nov.

C3F99341-FBE1-513E-B9BB-6C5D8548D745

http://zoobank.org/B01CEA82-FFB1-41DD-919C-6AC0B8D0EF59

Figs 14 , 15 , 38

##### Type material.

***Holotype*** ♂ (IZCAS-Ar 41012) and ***paratypes*** 17♀ (IZCAS-Ar 41013–41029), **China**: Taiwan Province, Nantou County, Ren’an Township, Hehuan Mountain, Yuanfeng Peak (24.11780°N, 121.23731°E; 2757 m), 2.VII.2013, S. Li and G. Zheng leg.; 1♂ (NHMSU-HA055) and 1♀ (NHMSU-HA055) used for sequencing, GenBank: MT991996 and MT991995, same data as for preceding.

##### Diagnosis.

*Crassignatha nantou* sp. nov. differs from other congeners, except *C. shunani* sp. nov., by the long, spiral embolus with a sharp, narrow tip and the separate bases of copulatory ducts (Fig. 15A, B, F, G). It is similar to *C. shunani* sp. nov. by the shape of the male palp and vulva, but the male can be distinguished by having a blunt cymbial tooth and a hook on the median apophysis vs. a sharp cymbial tooth and lack of a hook in the latter (Figs 15A, 29A); the female differs by the closer spermathecae and larger copulatory opening (Figs 15F, G, 29F, G).

**Figure 14.**
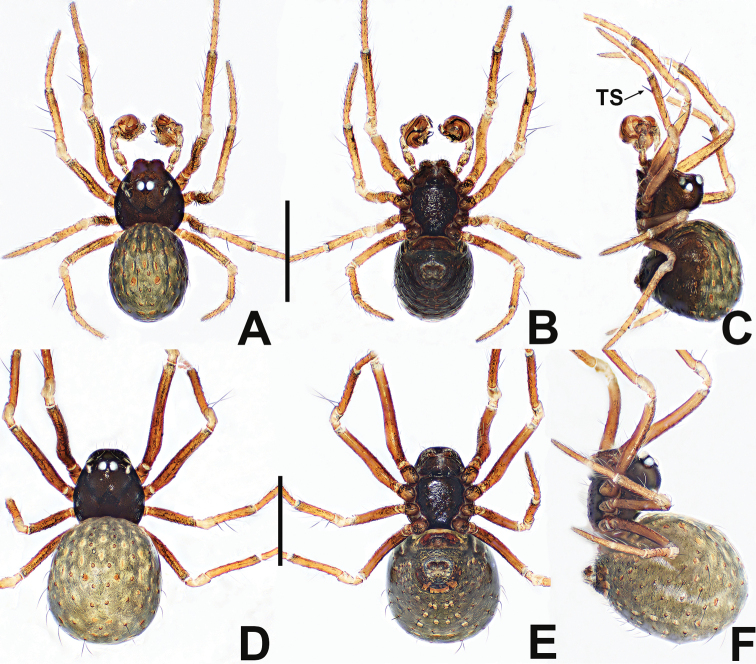
*Crassignatha nantou* sp. nov. **A** male habitus, dorsal **B** male habitus, ventral **C** male habitus, lateral **D** female habitus, dorsal **E** female habitus, ventral **F** female habitus, lateral. Scale bars: 0.50 mm (**A–F**).

##### Description.

**Male** (holotype). Total length 0.80. Carapace 0.36 long, 0.36 wide, 0.40 high. Clypeus 0.20 high. Sternum 0.24 long, 0.24 wide. Abdomen 0.52 long, 0.40 wide, 0.60 high. Length of legs: I 1.34 (0.42, 0.14, 0.32, 0.20, 0.26); II 1.10 (0.32, 0.12, 0.26, 0.16, 0.24); III 0.80 (0.22, 0.10, 0.16, 0.12, 0.20); IV 1.02 (0.32, 0.12, 0.22, 0.12, 0.24).

**Somatic characters** (Fig. 14A–C). ***Coloration***: carapace, sternum, chelicerae, endites, and labium dark brown. Abdomen blue-green with irregular sclerotized patches. ***Prosoma***: carapace nearly rounded, surface granular, with small sulci. Cephalic region strongly elevated. ALE protruded, PER recurved. Clypeus concave. Chelicerae covered with setae anteriorly. Labium semilunar, fused to sternum. Sternum heart shaped, flat, surface rough, truncated posteriorly. ***Legs***: tibia II with one clasping spur. ***Abdomen***: sub-elliptic dorsally, with lateral scuta. Spinnerets weakly sclerotized, with a circular plate.

**Figure 15.**
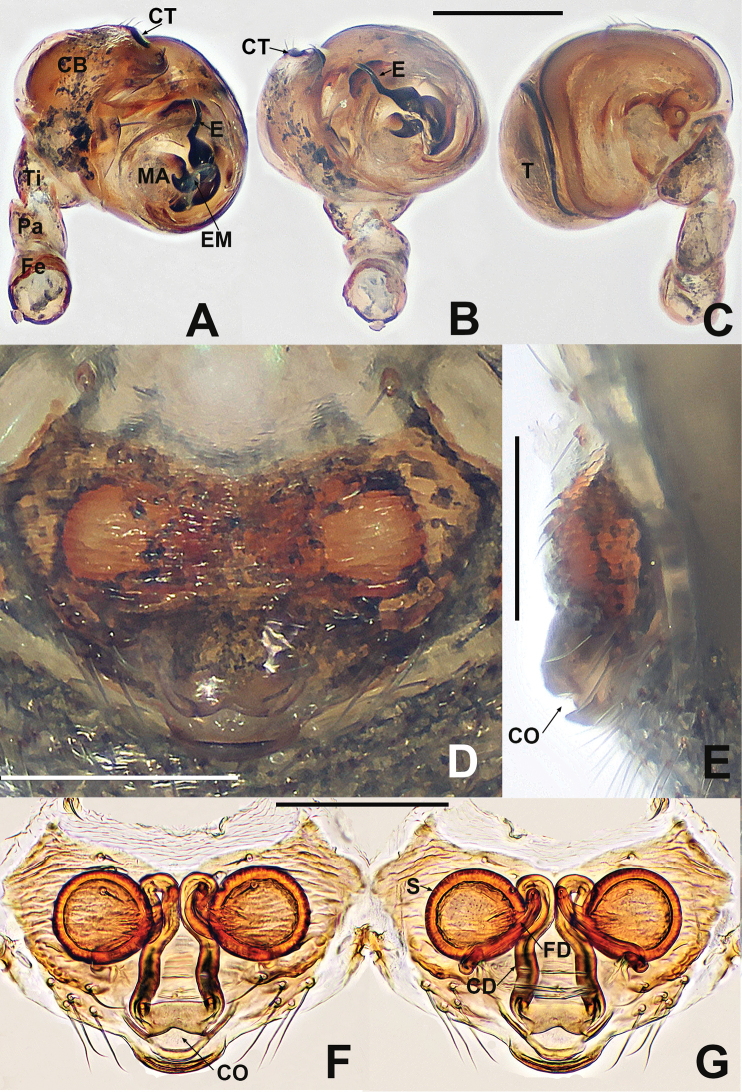
*Crassignatha nantou* sp. nov. **A** male palp, prolateral **B** male palp, ventral **C** male palp, retrolateral **D** epigyne, ventral **E** epigyne, lateral **F** vulva, ventral **G** vulva, dorsal. Scale bars: 0.10 mm (**A–G**).

***Palp*** (Fig. 15A–C): pale, weakly sclerotized. Cymbium large, with a few setae distally, cymbial tooth near distal margin. Tegulum large, smooth, and plump. Median apophysis disciform, with a hooked process on margin. Embolic membrane slender, laminar, translucent, arises near anterior part of median apophysis. Embolus long, flexible, spiraled, and wide basally, narrow distally.

**Female** (one of paratypes). Total length 1.08. Carapace 0.44 long, 0.40 wide, 0.40 high. Clypeus 0.16 high. Sternum 0.28 long, 0.28 wide. Abdomen 0.68 long, 0.60 wide, 0.80 high. Length of legs: I 1.68 (0.58, 0.16, 0.42, 0.24, 0.28); II 1.38 (0.46, 0.14, 0.34, 0.22, 0.22); III 1.00 (0.28, 0.12, 0.22, 0.16, 0.22); IV 1.24 (0.42, 0.14, 0.26, 0.18, 0.24).

**Somatic characters** (Fig. 14D–F). ***Coloration***: prosoma as in male. Abdominal color lighter than in male, dorsum lighter than venter. ***Prosoma***: carapace nearly pear shaped, sculptured, and granular. Cephalic region lower than in male. PER slightly recurved. Chelicerae fused near base, covered with setae anteriorly. Labium subtriangular, fused to sternum. Sternum as in male. ***Abdomen***: nearly globular dorsally, surface modified by sclerotized dots. Spinnerets weakly sclerotized, with a circular plate.

***Epigyne*** (Fig. 15D–G): epigynal area lightly sclerotized, with setae on lateral margins. Scape short, wide, copulatory opening located at its terminus, split into two labella. Internal structures more or less visible via translucent tegument. Paired spermathecae globose, separated by half their diameter. Fertilization ducts thin, starting at inside central margin of spermathecae, bent downward, twisted, and furcate at end. Copulatory ducts thick, connected to posterior margin of spermathecae, passing under the spermathecae, up into the center of vulva, deflexed to copulatory opening, their proximal base unfused.

##### Etymology.

The specific name is derived from the type locality; noun in apposition.

##### Distribution.

China (Taiwan) (Fig. 38).

#### Crassignatha nasalis

Taxon classificationAnimaliaAraneaeSymphytognathidae

Y. Lin & S. Li sp. nov.

0FC9F3B3-425E-5F25-B974-BA0664B98AEB

http://zoobank.org/7084BD59-EC9E-44F1-9DC2-F0E08117E7A7

Figs 16 , 17 , 38

##### Typ﻿e material.

***Holotype*** ♂ (NHMSU Ar 026) and ***paratypes*** 9♀ (NHMSU Ar 027–035), **China**: Sichuan Province, Luzhou City, Gulin County, Yuhua Township, Taoyuan Cave (27.98293°N, 105.99833°E; 910 m), 21.IV.2014, Y. Lin, H. Zhao, and Y. Li leg. 1♂ (NHMSU-HA041) and 1♀ (NHMSU-HA041) used for sequencing, GenBank: MT991992 and MT991991, same data as for preceding.

##### Diagnosis.

The male of *C. nasalis* sp. nov. is similar to that of *C. rostriformis* sp. nov. but can be distinguished by the sharp hook of the median apophysis and a straight cymbial tooth vs. a blunt hook of the median apophysis and a hook-like cymbial tooth (Figs 17A, B, 25A, B). The female is similar to *C. quanqu* in epigyne shape but differs from the latter by the indistinct scape and the copulatory ducts nearly forming a closed rhombic area at center of vulva vs. obvious scape and copulatory ducts not forming a closed area at center of vulva (Fig. 17E, F vs. Fig. 23D, E).

**Figure 16.**
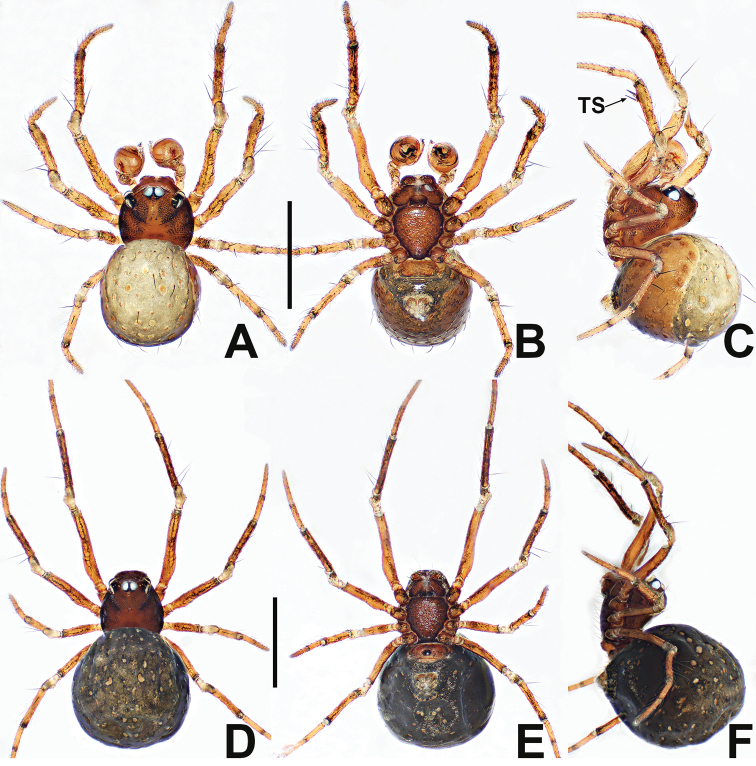
*Crassignatha nasalis* sp. nov. **A** male habitus, dorsal **B** male habitus, ventral **C** male habitus, lateral **D** female habitus, dorsal **E** female habitus, ventral **F** female habitus, lateral. Scale bars: 0.50 mm (**A–F**).

##### Description.

**Male** (holotype). Total length 0.80. Carapace 0.40 long, 0.32 wide, 0.44 high. Clypeus 0.12 high. Sternum 0.24 long, 0.20 wide. Abdomen 0.52 long, 0.44 wide, 0.56 high. Length of legs: I 1.10 (0.30, 0.14, 0.30, 0.16, 0.20); II 0.92 (0.24, 0.12, 0.24, 0.14, 0.18); III 0.72 (0.20, 0.10, 0.14, 0.10, 0.18); IV 0.82 (0.22, 0.10, 0.20, 0.12, 0.18).

**Somatic characters** (Fig. 16A–C). ***Coloration***: carapace, sternum, chelicerae, endites, and labium brown. Legs yellow-brown. Abdomen pale gray, ventrally darker than dorsally, with sclerotized dots and sparse setae; posterolateral scutum light brown. ***Prosoma***: carapace nearly rounded, thoracic center smooth, cephalic area and margins granulated and pitted, with two strong setae medially. Cephalic area strongly elevated. ALE protruded, PER strongly recurved. Clypeus concave. Chelicerae covered with setae anteriorly. Sternum almost heart shaped, slightly plump, truncated posteriorly, surface rough. ***Legs***: tibia II with two large clasping spurs. ***Abdomen***: sub-rounded dorsally. Spinnerets weakly sclerotized, with a circular plate.

**Figure 17.**
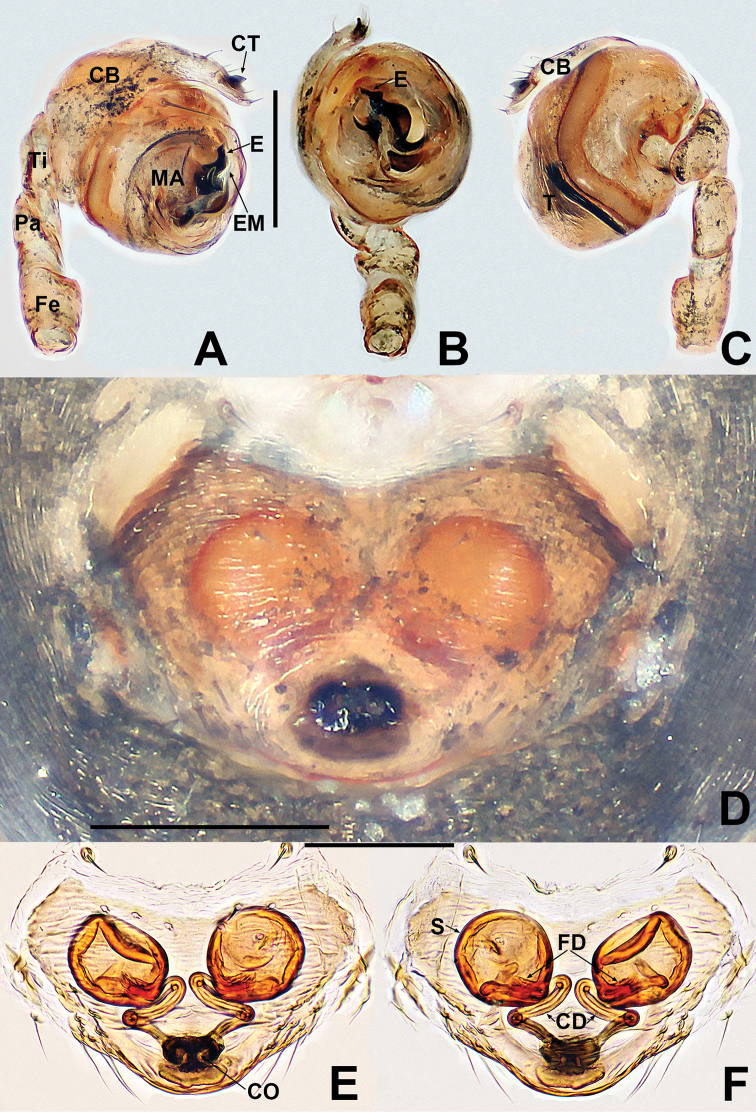
*Crassignatha nasalis* sp. nov. **A** male palp, prolateral **B** male palp, ventral **C** male palp, retrolateral **D** epigyne, ventral **E** vulva, ventral **F** vulva, dorsal. Scale bars: 0.10 mm (**A–F**).

***Palp*** (Fig. 17A–C): pale, weakly sclerotized. Cymbium with a few setae apically, cymbial tooth spur-like, located at distal terminus. Tegulum globose, smooth. Plate-like median apophysis with a spike-shaped distal process. Embolic membrane arises behind the median apophysis. Embolus short, rigid, basally constricted and mesally widened, distally forming an inverted Z-shape.

**Female** (one of paratypes). Total length 1.00. Carapace 0.40 long, 0.36 wide, 0.40 high. Clypeus 0.16 high. Sternum 0.24 long, 0.24 wide. Abdomen 0.68 long, 0.68 wide, 0.76 high. Length of legs: I 1.40 (0.46, 0.16, 0.32, 0.20, 0.26); II 1.18 (0.36, 0.14, 0.26, 0.18, 0.24); III 0.82 (0.20, 0.12, 0.16, 0.14, 0.20); IV 1.04 (0.30, 0.14, 0.24, 0.16, 0.20).

**Somatic characters** (Fig. 16D–F). ***Coloration***: prosoma and legs as in male. Abdomen dark, with tiny light yellow dots. ***Prosoma***: carapace nearly pear shaped, ocular arrangement and modification as in male. Cephalic area lower than in male. PER straight. Mouthparts and sternum as in male. ***Abdomen***: globular dorsally. Spinnerets weakly sclerotized.

***Epigyne*** (Fig. 17D–F): epigynal area distinctly sclerotized, with a few setae. Scape short, strongly sclerotized. Copulatory openings separated, resembling a pig snout. Vulva visible via translucent tegument. Paired spermathecae separated by less than ½ their diameter. Fertilization ducts originate inside posterior edge of spermathecae, curving toward center of spermathecae. Copulatory ducts connected to dorsal center surface of spermathecae, twisting 3 ×, then forming a nearly closed rhombic area in center of vulva.

##### Etymology.

The specific epithet is a Latin adjective (= nasal) and refers to the shape of the copulatory openings of the epigyne.

##### Distribution.

China (Sichuan) (Fig. 38).

#### Crassignatha pianma

Taxon classificationAnimaliaAraneaeSymphytognathidae

Miller, Griswold & Yin, 2009

4F2810C2-77BD-581A-9836-6F07FE52D313

Figs 18 , 19 , 38

Crassignatha pianma Miller et al., 2009: 70, figs 67I, 74A–E, 75A, B, 76A, B, 77A–F, 78A–D, 79A, B (♂♀).

##### Type material.

***Holotype*** ♂ (HNU-CASENT 9022600) and ***paratypes*** 2♂ 6♀ (HNU-CASENT 9022360), **China**: Yunnan Province, Lushui County, Pianma Township, Changyan River, 9.3 km ESE Pianma, mixed broadleaf deciduous and evergreen forest, dusting small webs near ground in forest understory (25.99363°N, 98.66651°E; 2470 m), 13–15.V.2005, C. Griswold leg. Examined.

**Figure 18.**
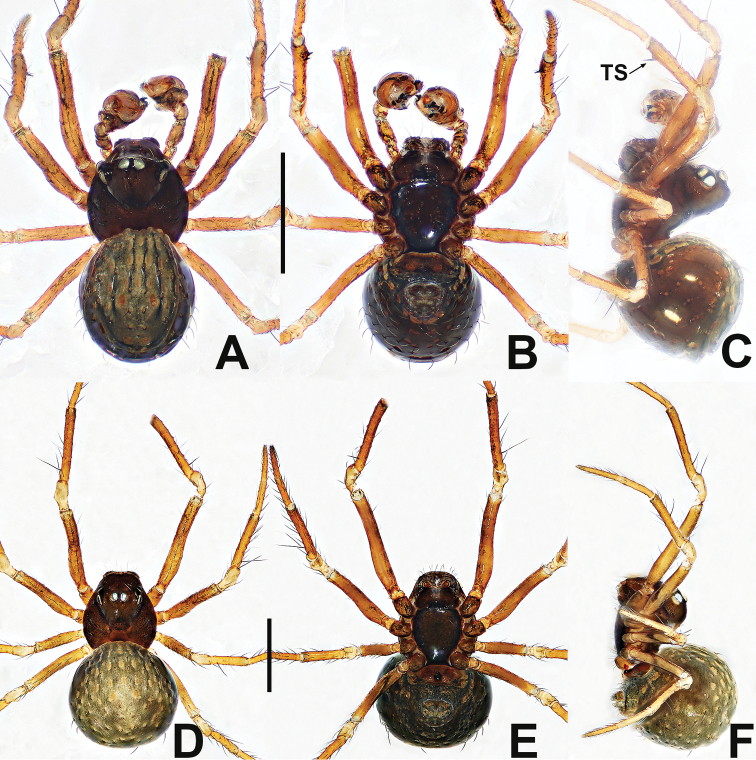
*Crassignatha pianma* Miller, Griswold & Yin, 2009 **A** male habitus, dorsal **B** male habitus, ventral **C** male habitus, lateral **D** female habitus, dorsal **E** female habitus, ventral **F** female habitus, lateral. Scale bars: 0.50 mm (**A–F**).

##### Other material examined.

21♂ 93♀ (NHMSU-HA113), **China**: Yunnan Province, Lushui County, Pianma Town, Changyan River, mixed broadleaf deciduous and evergreen forest, dusting small webs near ground in forest (25.99363°N, 98.66651°E; 2470 m), 10.VIII.2018, Y. Lin et al. leg.; 1♂ (NHMSU-HA113) and 1♀ (NHMSU-HA113) used for sequencing, GenBank: MT992009 and MT992008, same data as for preceding.

##### Diagnosis.

**Figure 19.**
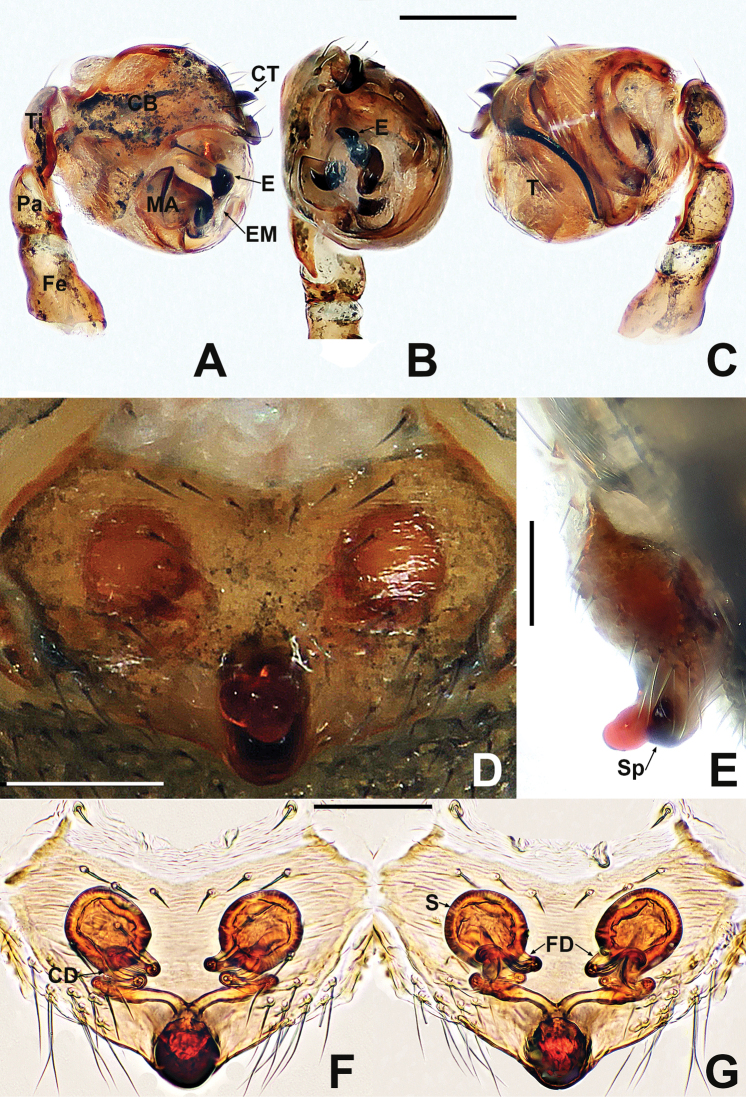
*Crassignatha pianma* Miller, Griswold & Yin, 2009 **A** male palp, prolateral **B** male palp, ventral **C** male palp, retrolateral **D** epigyne, ventral **E** epigyne, lateral **F** vulva, ventral **G** vulva, dorsal. Scale bars: 0.10 mm (**A–G**).

The male of *C. pianma* is similar to *C. quanqu* in the form of the palp but can be distinguished from the latter by the subquadrate median apophysis with a hooked process, rather than a subtriangular median apophysis with a truncated process (Figs 19A, B, 23A, B). The female of *C. pianma* seems closest to that of *C. bangbie* sp. nov. but differs by the more slender, more twisted copulatory ducts than in the latter, and the smaller copulatory openings (Figs 19E–G, 3E–G).

##### Description.

See Miller et al. (2009).

##### Distribution.

China (Yunnan) (Fig. 38).

#### Crassignatha quadriventris

Taxon classificationAnimaliaAraneaeSymphytognathidae

(Lin & Li, 2009) comb. nov.

EAF96456-8E8A-5479-9AD3-4A848E56144A

Figs 20 , 21 , 38

Patu quadriventris Lin & Li, 2009: 55, figs 7A, B, 8A, B, 9A–E, 10A, B (♂♀).

##### Type material.

***Holotype*** ♂ and ***paratypes*** 2♂ 2♀ (IZCAS), **China**: Hainan Province, Wuzhishan City, Mt. Wuzhishan Nature Reserve (18.90000°N, 109.65000°E), 9.VIII.2007, S. Li and C. Wang leg.; ***paratype*** 1♀ (IZCAS), **China**: Hainan Province, Qiongzhong County, Limushan Nature Reserve (19.18333°N, 109.73333°E; 655 m), 12.VIII.2007, S. Li and C. Wang leg. Examined.

**Figure 20.**
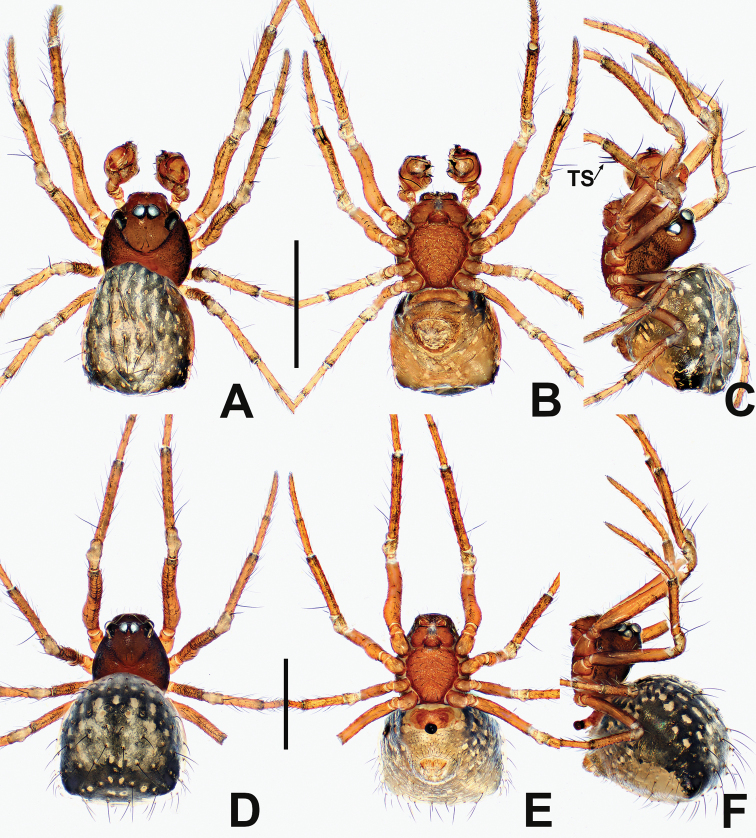
*Crassignatha quadriventris* (Lin & Li, 2009) comb. nov. **A** male habitus, dorsal **B** male habitus, ventral **C** male habitus, lateral **D** female habitus, dorsal **E** female habitus, ventral **F** female habitus, lateral. Scale bars: 0.50 mm (**A–F**).

##### Other material examined.

1♂ 4♀ (IZCAS-Ar 41030–41034), **China**: Hainan Province, Dongfang City, Donghe Town, Nanlang Village, at the foot of Exianling Mountain (19.00633°N, 109.08383°E; 214 m), 16.XII.2014, Q. Zhao and L. Shao leg.; 1♀ (NHMSU-HA025) used for sequencing, GenBank: MT991990, same data as for preceding.

##### Diagnosis.

**Figure 21.**
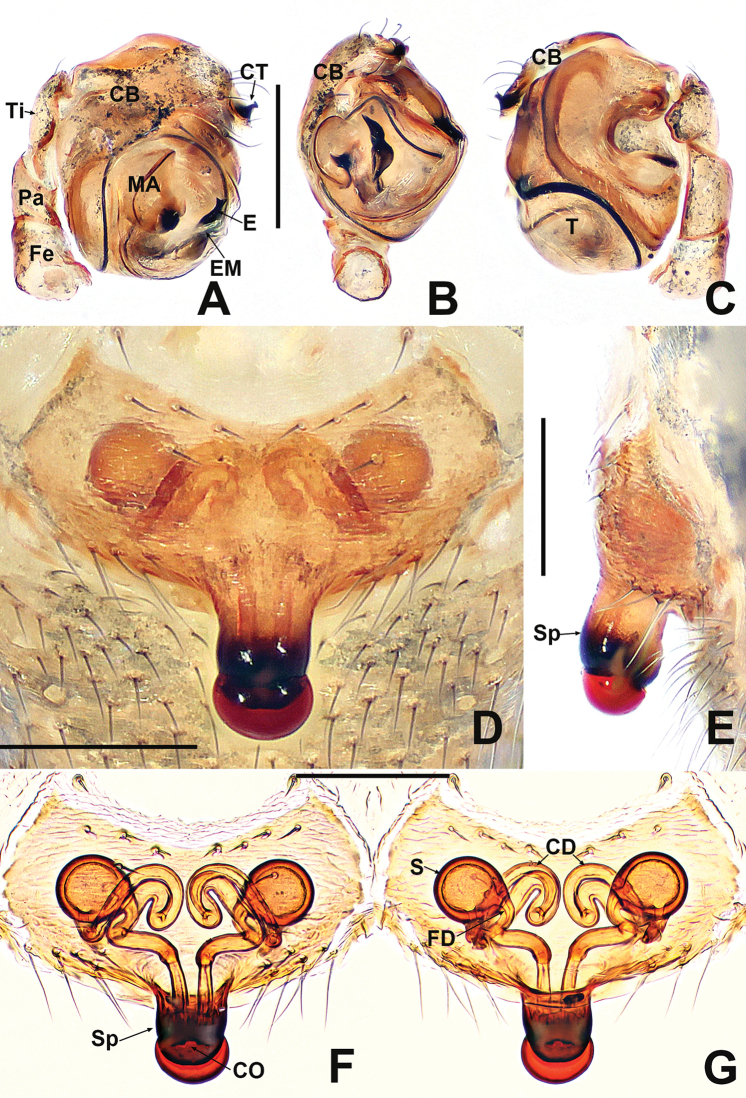
*Crassignatha quadriventris* (Lin & Li, 2009) comb. nov. **A** male palp, prolateral **B** male palp, ventral **C** male palp, retrolateral **D** epigyne, ventral **E** epigyne, lateral **F** vulva, ventral **G** vulva, dorsal. Scale bars: 0.10 mm (**A–G**).

This species differs from other congeneric species except *C. baihua* sp. nov. by the short, stiff embolus (Fig. 21A, B). It is most similar to *C. baihua* sp. nov. in the form of the palp and the vulva configuration but can be easily distinguished by the sloped embolic apex, rather than flat as in the latter, and by having six twists of the copulatory ducts, rather than four in the latter (Figs 21A, B, F, G, 2A, B, F, G).

##### Description.

**Male** (IZCAS-Ar 41030). Total length 0.80. Carapace 0.36 long, 0.36 wide, 0.40 high. Clypeus 0.16 high. Sternum 0.28 long, 0.24 wide. Abdomen 0.52 long, 0.48 wide, 0.52 high. Length of legs: I 1.34 (0.42, 0.14, 0.34, 0.20, 0.24); II 1.10 (0.30, 0.14, 0.26, 0.16, 0.24); III 0.90 (0.24, 0.12, 0.18, 0.14, 0.22); IV 1.02 (0.30, 0.12, 0.22, 0.18, 0.20).

**Somatic characters** (Fig. 20A–C). ***Coloration***: prosoma brown. Legs pale brown. Abdomen dorsally dark, with pale stripes and speckles, ventrally pale brown. ***Prosoma***: carapace sub-rounded, granular, sculptured, with two cephalic setae. Cephalic area elevated. Clypeus concave. ALE protruded. PER strongly recurved. Chelicerae covered with few setae anteriorly. Labium tongue shaped, wider than long. Sternum heart shaped, surface rough, slightly plump, truncated posteriorly. ***Legs***: tibia II with two clasping spurs. ***Abdomen***: anteriorly round, nearly square posteriorly, with sparse, long setae, lateral scutum distinct. Spinnerets slightly sclerotized, surrounded by a circular plate.

***Palp*** (Fig. 21A–C): tibia with four distal, dorsal, short setae. Cymbium bears a few distal setae. Apical cymbial tooth hook shaped. Tegulum broad, smooth, globular. Plate-like median apophysis with a sclerotized, longitudinal central ridge line and a prolaterally odontoid process. Embolic membrane arises near anterior part of median apophysis. Embolus short, stiff, basally constricted, mesally wide, distally coracoid.

**Female** (IZCAS-Ar 41031). Total length 1.04. Carapace 0.44 long, 0.40 wide, 0.40 high. Clypeus 0.14 high. Sternum 0.28 long, 0.28 wide. Abdomen 0.72 long, 0.68 wide, 0.80 high. Length of legs: I 1.72 (0.60, 0.18, 0.40, 0.26, 0.28); II 1.42 (0.44, 0.16, 0.34, 0.20, 0.28); III 0.98 (0.26, 0.10, 0.22, 0.16, 0.24); IV 1.18 (0.34, 0.12, 0.28, 0.18, 0.26).

**Somatic characters** (Fig. 20D–F). ***Coloration***: prosoma and legs as in male. Abdomen dorsally darker than in male, ventrally lighter. ***Prosoma***: modification and arrangement of eyes as in male, cephalic area lower than in male. Clypeus slightly concave. Mouthparts and sternum as in male. ***Abdomen***: anteriorly round and posteriorly square, surface bears sparse, long setae, lateral scutum and circular plate absent. Spinnerets slightly sclerotized.

***Epigyne*** (Fig. 21D–F): epigynal area weakly sclerotized, bears a few setae. Scape developed, protruded, longer than wide. Copulatory openings located at terminus. Internal structures faintly visible via translucent tegument. Paired spermathecae globose, widely separated by at least 1.5× their diameter. Fertilization ducts originating from the lower inside margin of spermathecae, bent downwards and laterally. Copulatory ducts long, connected to dorsal surface of spermathecae, from below spermathecae to the center of vulva, making six bends, then reaching copulatory openings.

##### Taxonomic justification.

The shape of the male palps, the configuration of the epigyne, the modified carapace, and the male abdominal scutum and clasping spurs on tibia II leave no doubt that this species is a member of *Crassignatha* and not *Patu*. Therefore, we propose a new combination, *C. quadriventris* (Lin & Li, 2009) comb. nov., transferring it from *Patu*.

##### Distribution.

China (Hainan) (Fig. 38).

#### Crassignatha quanqu

Taxon classificationAnimaliaAraneaeSymphytognathidae

Miller, Griswold & Yin, 2009

A2191A75-68E9-5388-9150-98952E4338F5

Figs 22 , 23 , 38

Crassignatha quanqu Miller et al., 2009: 72, figs 76E–G, 78F, 79E, F, 83A–E, 84A, B, 85A–F (♂♀).

##### Type material.

***Holotype*** ♂ (HNU-CASENT 9029323) and ***paratypes*** 1♂ 1♀ (HNU-CASENT 9022388), **China**: Yunnan Province, Longling County, Zhen’an Township, Bangbie Village, at stream at km 6.8 on Route S317, shady embankments along stream, dusting webs in understory (24.81333°N, 98.83280°E, 1552.5 ± 7.5 m), 24.V.2005, C. Griswold leg. Examined.

**Figure 22.**
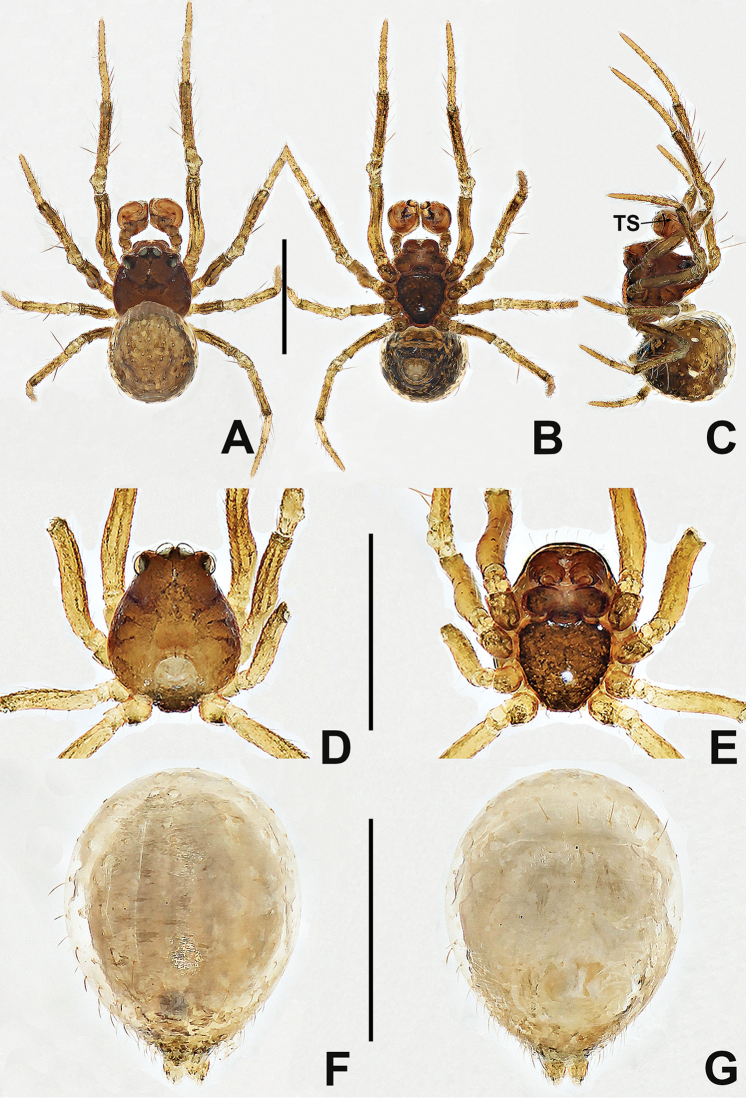
*Crassignatha quanqu***A** male habitus, dorsal **B** male habitus, ventral **C** male habitus, lateral **D** female prosoma, dorsal **E** female prosoma, ventral **F** female abdomen, dorsal **G** female abdomen, ventral. Scale bars: 0.50 mm (**A–G**).

##### Diagnosis.

**Figure 23.**
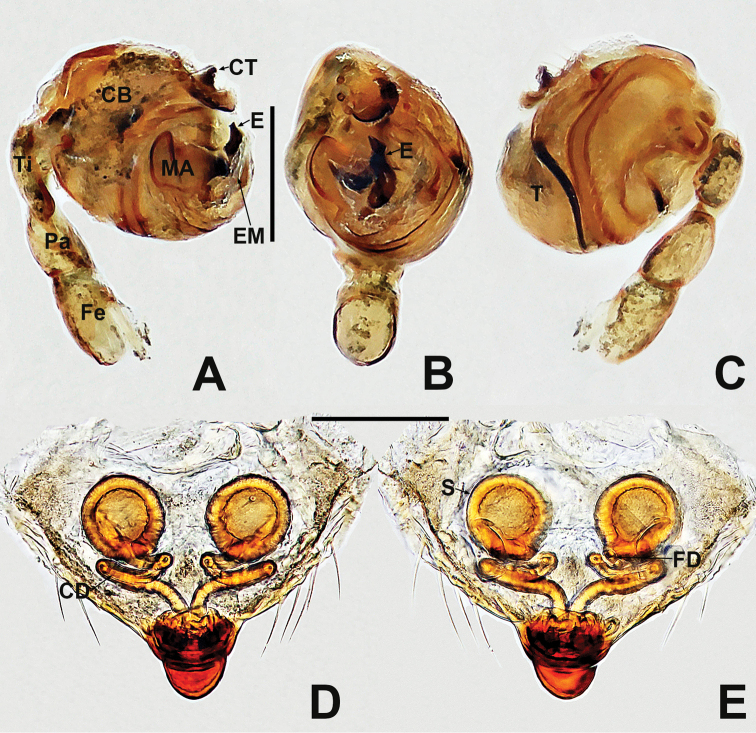
*Crassignatha quanqu***A** male palp, prolateral **B** male palp, ventral **C** male palp, retrolateral **D** vulva, ventral **E** vulva, dorsal. Scale bars: 0.10 mm (**A–E**).

The male of *Crassignatha quanqu* is similar to *C. gucheng* sp. nov. but can be distinguished by the median apophysis with two tapered distal processes and the details of the palp (Fig. 23A–C vs. Fig. 10A–C). The female of *C. quanqu* is similar to *C. yinzhi* and *C. thamphra* sp. nov. in the vulva configuration, but differs by the nearly vertical connection of copulatory ducts and copulatory openings, rather than connected diagonally as in the latter two, and differs by the fertilization ducts starting at the posterior margin of the spermathecae, rather than the inside medial margin as in the latter two (Fig. 23D, E vs. Figs 32F, G, 37F, G).

##### Description.

See Miller et al. (2009).

##### Distribution.

China (Yunnan) (Fig. 38).

#### Crassignatha rostriformis

Taxon classificationAnimaliaAraneaeSymphytognathidae

Y. Lin & S. Li sp. nov.

EFC94EA1-FA6F-5EF2-943F-B22F62B2B905

http://zoobank.org/3F86509B-A293-4591-85EF-E84F270C91AB

Figs 24 , 25 , 38

##### Type material.

***Holotype*** ♂ (NHMSU Ar 036) and ***paratypes*** 2♂ 34♀ (NHMSU Ar 037–072), **China**: Yunnan Province, Wenshan Prefecture, Xichou County, Dongma Township, Xianrendong Village, Xianren Cave (23.50193°N, 104.86810°E; 1326 m), 6.VIII.2010, Z. Yao, X. Wang and C. Wu leg.; 2♂ 16♀ (NHMSU-HA078), 3♂ 2♀ (NHMSU-HA103), same data as holotype; 1♂ (NHMSU-HA079) and 1♀ (NHMSU-HA079) used for sequencing, GenBank: MT991998 and MT991997, same data as for preceding.

**Figure 24.**
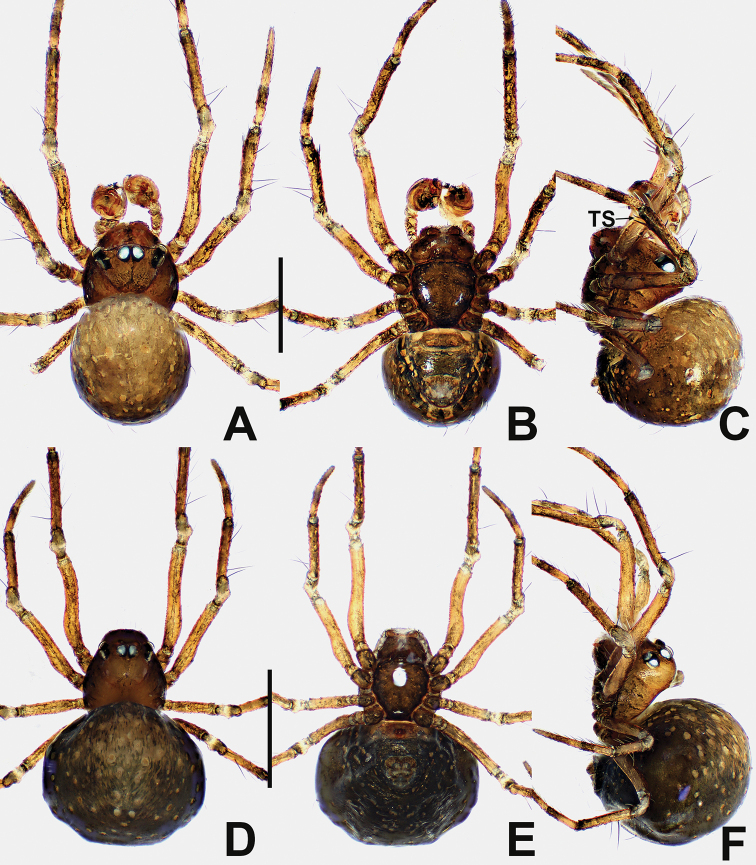
*Crassignatha rostriformis* sp. nov. **A** male habitus, dorsal **B** male habitus, ventral **C** male habitus, lateral **D** female habitus, dorsal **E** female habitus, ventral **F** female habitus, lateral. Scale bars: 0.50 mm (**A–F**).

##### Diagnosis.

**Figure 25.**
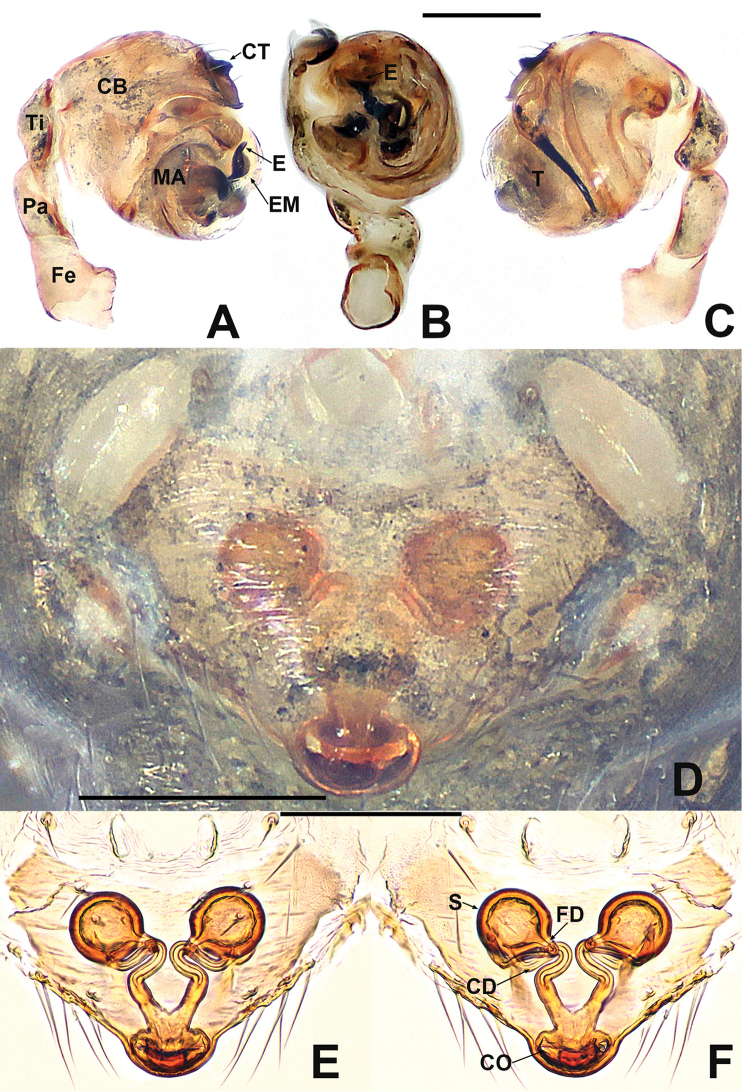
*Crassignatha rostriformis* sp. nov. **A** male palp, prolateral **B** male palp, ventral **C** male palp, retrolateral **D** epigyne, ventral **E** vulva, ventral **F** vulva, dorsal. Scale bars: 0.10 mm (**A–F**).

The male of *C. rostriformis* sp. nov. is similar to *C. nasalis* sp. nov. in the form of the palp but differs from the latter by the large, hooked cymbial tooth and the wider embolic base (Fig. 25B vs. Fig. 17A, B). The female is most similar to that of *C. mengla* sp. nov. in the vulva configuration but can be easily distinguished by spermathecae separated by less than their diameter, and the low second turn of the copulatory duct, rather than wide intervals of spermathecae and the high second turn of the copulatory duct (Fig. 25D–F vs. Fig. 13F, G).

##### Description.

**Male** (holotype). Total length 0.64. Carapace 0.28 long, 0.28 wide, 0.32 high. Clypeus 0.10 high. Sternum 0.20 long, 0.20 wide. Abdomen 0.40 long, 0.36 wide, 0.48 high. Length of legs: I 1.04 (0.32, 0.12, 0.24, 0.16, 0.20); II 0.84 (0.22, 0.12, 0.20, 0.12, 0.18); III 0.66 (0.20, 0.10, 0.12, 0.10, 0.14); IV 0.80 (0.26, 0.10, 0.16, 0.12, 0.16).

**Somatic characters** (Fig. 24A–C). ***Coloration***: prosoma dark brown, ventrally darker than dorsally. Legs brown, with black pigmentation. Abdomen dark, laterally and ventrally darker than dorsally, with light brown speckles. ***Prosoma***: carapace sub-rounded, surface sculptured. Cephalic area elevated. Clypeus concave. ALE slightly protruded. PER recurved. Mouthparts distinctly sclerotized. Labium nearly semilunar. Sternum scutiform, surface subtly textured, slightly plump, truncated posteriorly. ***Legs***: tibia II with two clasping spurs. ***Abdomen***: rounded dorsally, abdominal lateral scutum weakly sclerotized, circular plate absent. Spinnerets tiny.

***Palp*** (Fig. 25A–C): tibia as long as patella. Cymbium wider than femur, bears some distal setae, with a dorsal hooked tooth near distal margin. Tegulum plump and globular. Disciform median apophysis with an odontoid prolateral process. Embolic membrane arises from behind median apophysis. Embolus short, rigid, basally wide, mesally and distally narrow.

**Female** (one of paratypes). Total length 0.92. Carapace 0.40 long, 0.36 wide, 0.36 high. Clypeus 0.12 high. Sternum 0.24 long, 0.24 wide. Abdomen 0.60 long, 0.72 wide, 0.68 high. Length of legs: I 1.36 (0.46, 0.12, 0.32, 0.22, 0.24); II 1.12 (0.38, 0.14, 0.26, 0.16, 0.18); III 0.82 (0.22, 0.12, 0.14, 0.16, 0.18); IV 1.02 (0.30, 0.12, 0.24, 0.16, 0.20).

**Somatic characters** (Fig. 24D–F). ***Coloration***: prosoma as in male. Abdomen darker than in male. ***Prosoma***: carapace nearly pear shaped, weakly granular. Cephalic area lower than in male. PER slightly recurved. Mouthparts and sternum as in male. ***Abdomen***: nearly globose, dorsally speckled. Spinnerets weakly sclerotized.

***Epigyne*** (Fig. 25D–F): slightly sclerotized, with a few setae at lateral margins. Scape short, slightly protruded. Copulatory openings large, flat, beak shaped, located at the terminus of scape. Internal structures faintly visible via translucent tegument. Spermathecae globose, separated by ~0.8× their diameter. Fertilization ducts starting at inside posterior margin of spermathecae and bending below the venter of spermathecae. Copulatory ducts relatively long, connected to lower dorsal surface of spermathecae, bypassing around spermathecae, forming three bends, fusing before copulatory openings.

##### Etymology.

The specific epithet is derived from the Latin adjective *rostriformis* (rostriform), in reference to the shape of the copulatory openings.

##### Distribution.

China (Yunnan) (Fig. 38).

#### Crassignatha shiluensis

Taxon classificationAnimaliaAraneaeSymphytognathidae

(Lin & Li, 2009) comb. nov.

8D40BBEC-E760-5DB4-BD9F-DC5A194B063D

Figs 26 , 27 , 38

Patu shiluensis Lin & Li, 2009: 59, figs 11A, B, 12A, B, 13A–D (♂♀).

##### Type material.

***Holotype*** ♂ and ***paratypes*** 4♂ 9♀ (IZCAS), **China**: Hainan Province, Changjiang Lizu Autonomous County, Shilu Town, in leaf litter in rainforest (19.20000°N, 109.06667°E), 22.III.2005, Y. Tong, Y. Song and X. Han leg. Examined.

**Figure 26.**
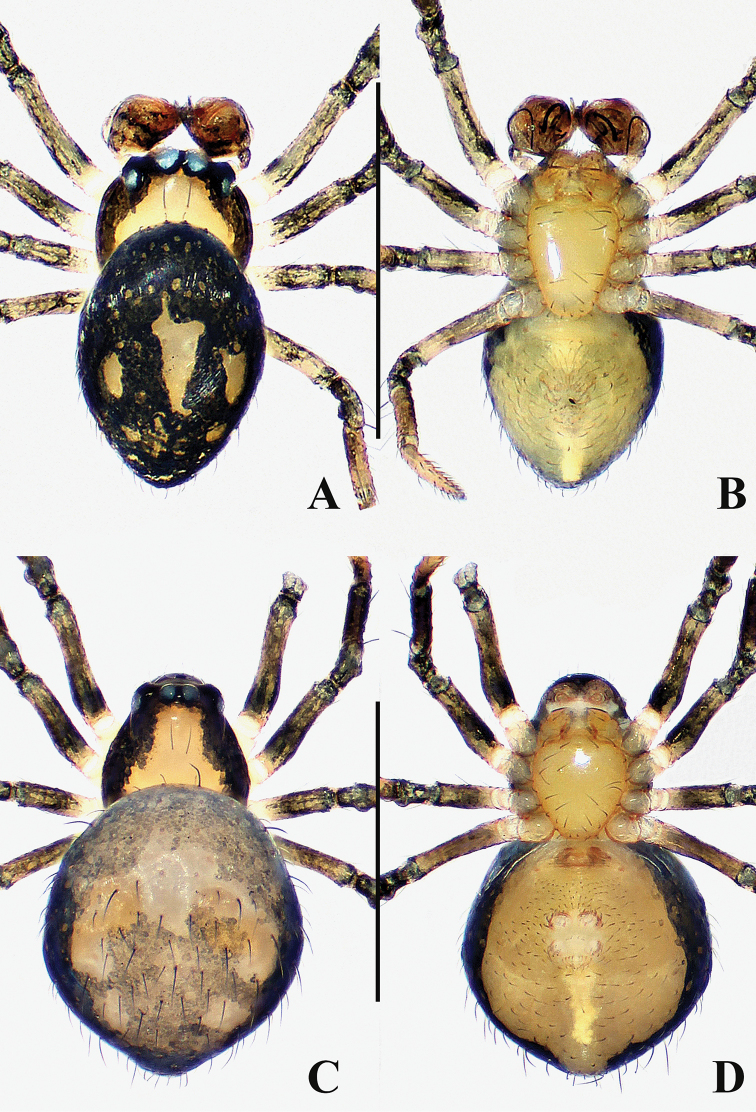
*Crassignatha shiluensis* (Lin & Li, 2009) comb. nov. **A** male habitus, dorsal, **B** male habitus, ventral **C** female habitus, dorsal **D** female habitus, ventral. Scale bars: 0.50 mm (**A–D**).

##### Other material examined.

3♂ 8♀ (NHMSU-HA081), **China**: Yunnan Province, Mengla County, Menglun Town, Xishuangbanna Tropical Botanic Garden, tropical rainforest (21.917°N, 101.275°E; 558 m), 5.X.2017, Y. Lin and Y. Li leg.; 1♂ juvenile (NHMSU-HA081) and 1♀ (NHMSU-HA081) used for sequencing, GenBank: MT992002 and MT992001, same data as for preceding; 1♂ 3♀ (NHMSU-HA059), **China**: Yunnan Province, Menglun Nature Reserve, secondary tropical seasonal moist forest (21.91197°N, 101.28233°E; 645 ± 17 m), 5–12.I.2007, G. Zheng leg.

##### Diagnosis.

**Figure 27.**
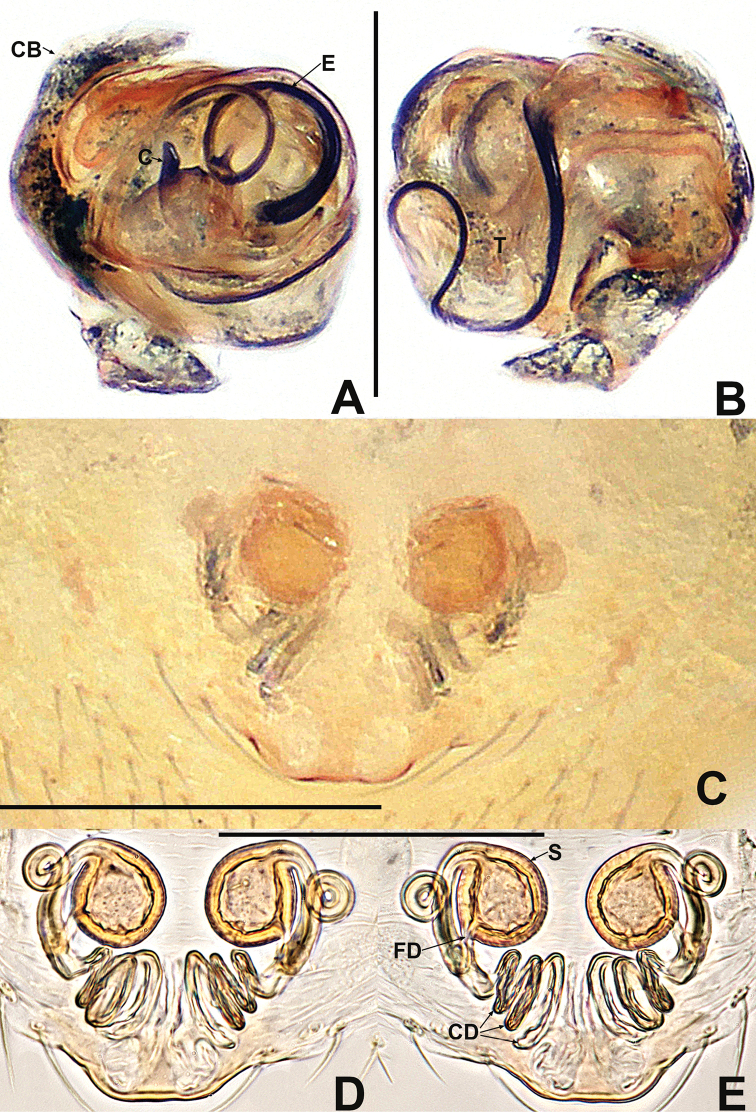
*Crassignatha shiluensis* (Lin & Li, 2009) comb. nov. **A** male palp, prolateral **B** male palp, retrolateral **C** epigyne, ventral **D** vulva, ventral **E** vulva, dorsal. Scale bars: 0.10 mm (**A–E**).

This species differs from all other species of *Crassignatha* by the long embolus coiling into two loops (Fig. 27A) and by the long copulatory ducts connected to the anterolateral margin of the spermathecae, coiled into three loops below spermathecae (Fig. 27D, E). Its dorsoventral dichroism is also a prominent feature (Fig. 26A–D).

##### Description.

See Lin and Li (2009).

##### Taxonomic justification.

A series of combinations: the form of the male palp and the configuration of the epigyne, the chelicerae fused at the base, and the male clasping setae distoventrally on tibia II suggest that this species is more similar to *Crassignatha* than *Patu*. It shares homologous characters of *Crassignatha*, such as a large median apophysis on a slightly oblate male palpal bulb and globular spermathecae rather than a nearly oviform male palpal bulb and claviform spermathecae as in *Patu*. Thus, we propose a new combination, *Crassignatha shiluensis* (Lin & Li, 2009) comb. nov., transferring it from *Patu*.

##### Distribution.

China (Hainan, Yunnan) (Fig. 38).

#### Crassignatha shunani

Taxon classificationAnimaliaAraneaeSymphytognathidae

Y. Lin & S. Li sp. nov.

62BD0CF8-12DE-5C68-869D-6C0F41D2D108

http://zoobank.org/099E51E5-0B4A-4433-8BFA-995B8FB3E784

Figs 28 , 29 , 38

##### Type material.

***Holotype*** ♂ (NHMSU Ar 073) and ***paratypes*** 4♂ 49♀ (NHMSU Ar 074–126), **China**: Sichuan Province, Luzhou City, Gulin County, Jianzhu Township, Wenyi Village, Dahei Cave (28.06134°N, 105.58015°E; 852 m), 23.IV.2014, Y. Lin, H. Zhao and J. Wu leg.; 1♂ juvenile (NHMSU-HA046) and 1♀ (NHMSU-HA046) used for sequencing, GenBank: MT991994 and MT991993, same data as for preceding.

##### Diagnosis.

This species is similar to *C. nantou* sp. nov. in the form of the male palp and the vulva configuration but can be distinguished by having a sharp cymbial tooth and lacking a hooked process on the median apophysis (Fig. 29A vs. Fig. 15A), and by the more widely separated spermathecae and lower inflection point of the copulatory ducts in the center of the vulva (Figs 29F, 15G).

**Figure 28.**
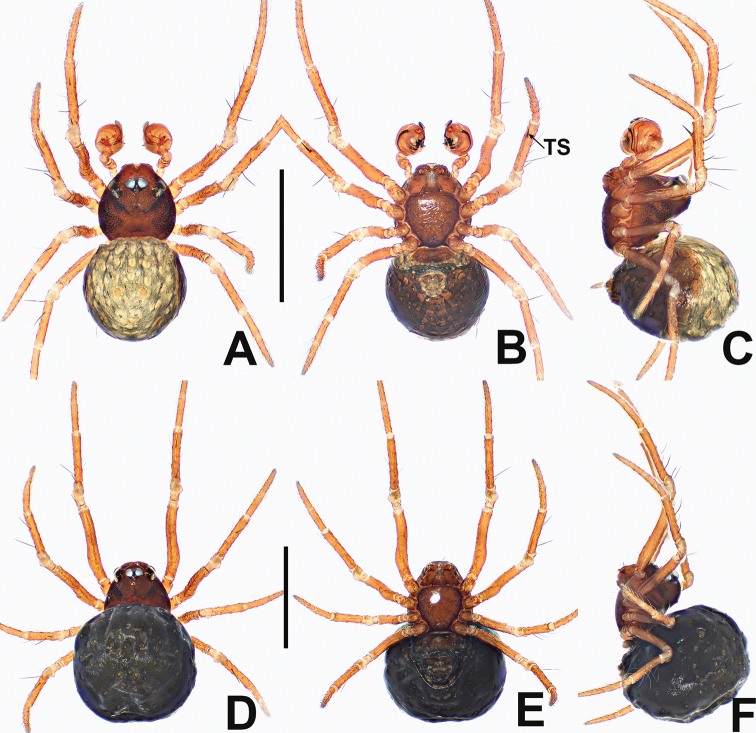
*Crassignatha shunani* sp. nov. **A** male habitus, dorsal **B** male habitus, ventral **C** male habitus, lateral **D** female habitus, dorsal **E** female habitus, ventral **F** female habitus, lateral. Scale bars: 0.50 mm (**A–F**).

##### Description.

**Male** (holotype). Total length 0.68. Carapace 0.32 long, 0.28 wide, 0.32 high. Clypeus 0.12 high. Sternum 0.20 long, 0.20 wide. Abdomen 0.40 long, 0.40 wide, 0.48 high. Length of legs: I 1.02 (0.32, 0.12, 0.24, 0.16, 0.18); II 0.88 (0.24, 0.12, 0.20, 0.16, 0.16); III 0.66 (0.18, 0.10, 0.12, 0.10, 0.16); IV 0.76 (0.22, 0.10, 0.16, 0.12, 0.16).

**Somatic characters** (Fig. 28A–C). ***Coloration***: prosoma brown. Legs pale brown. Abdomen grayish yellow dorsally, dark brownish laterally and ventrally, with weakly sclerotized dots dorsally. ***Prosoma***: carapace sub-rounded, cephalic and thoracic area granular, thoracic center and clypeus smooth. Cephalic area strongly elevated. Clypeus concave. ALE protruded. PER distinctly recurved. Chelicerae bears short setae anteriorly. Labium subtriangular. Sternum heart shaped, surface textured, slightly swollen, truncated posteriorly. ***Legs***: tibia II with two clasping spurs. ***Abdomen***: nearly globular, posterolateral scutum weakly sclerotized. Spinnerets without circular plate.

**Figure 29.**
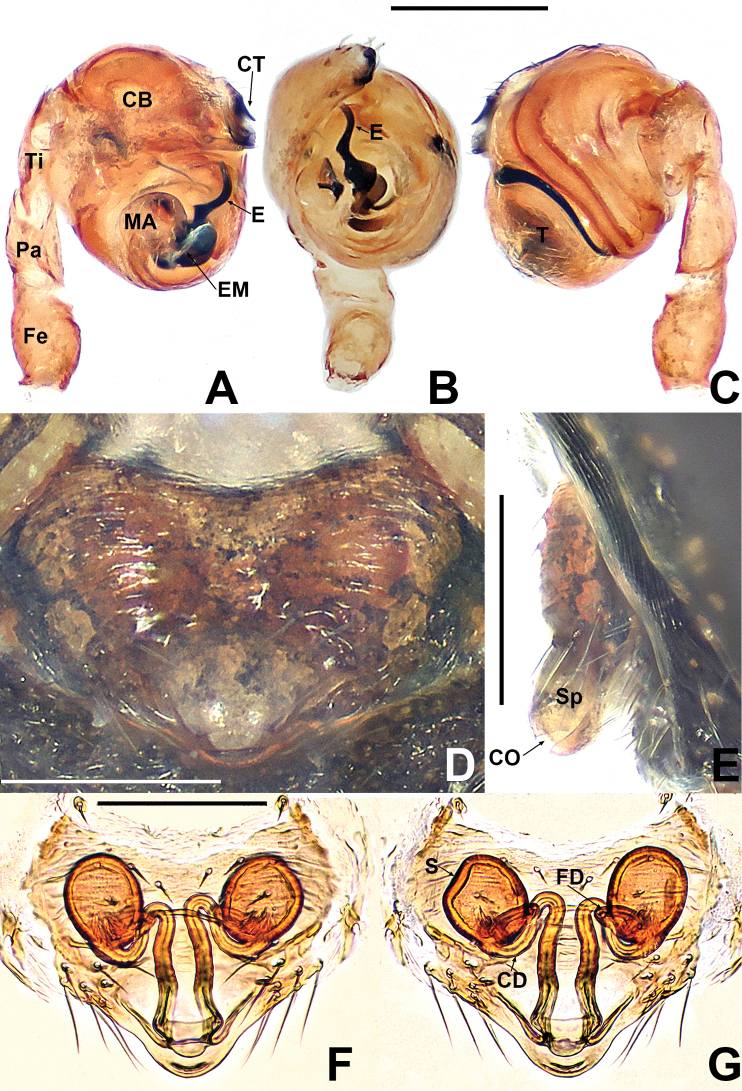
*Crassignatha shunani* sp. nov. **A** male palp, prolateral **B** male palp, ventral **C** male palp, retrolateral **D** epigyne, ventral **E** epigyne, lateral **F** vulva, ventral **G** vulva, dorsal. Scale bars: 0.10 mm (**A–G**).

***Palp*** (Fig. 29A–C): tibia laminar, subequal to patella in length. Cymbium with some setae at distal margin, horn-shaped cymbial tooth near cymbial apex. Tegulum globose, swollen, surface rugose. Nearly rounded median apophysis prolaterally on bulb, with short, straight distal process. Embolic membrane arises beside median apophysis. Embolus long, spiral, basally and mesally wide, distally narrow and bent.

**Female** (one of paratypes). Total length 0.80. Carapace 0.36 long, 0.32 wide, 0.32 high. Clypeus 0.12 high. Sternum 0.24 long, 0.20 wide. Abdomen 0.56 long, 0.60 wide, 0.64 high. Length of legs: I 1.12 (0.38, 0.12, 0.26, 0.18, 0.18); II 0.94 (0.26, 0.12, 0.22, 0.14, 0.20); III 0.74 (0.18, 0.10, 0.16, 0.12, 0.18); IV 0.88 (0.26, 0.12, 0.18, 0.12, 0.20).

**Somatic characters** (Fig. 28D–F). ***Coloration***: prosoma and legs as in male. Abdomen black, with sclerotized dots dorsally. ***Prosoma***: carapace pear shaped, surface modification and arrangement of eyes as in male. Cephalic area lower than in male. Mouthparts and sternum as in male. ***Abdomen***: globular. Spinnerets tiny, lacking circular plate.

***Epigyne*** (Fig. 29D–G): epigynal area slightly sclerotized. Scape protruded, as wide as long. Copulatory openings located at terminus of scape. Internal structures faintly visible via translucent tegument. Spermathecae strongly sclerotized, separated by their diameter. Fertilization ducts originating posteromedially from spermathecae. Copulatory ducts long, connected to posterior margin of spermathecae, curved upward and inward to center of vulva, then turned sharply downward to copulatory openings. The base of copulatory ducts unfused.

##### Etymology.

The specific name is from the Chinese pinyin *shŭ nán*, referring to the collection locality of this new spider species from southern Sichuan; noun in apposition.

##### Distribution.

China (Sichuan) (Fig. 38).

#### Crassignatha si

Taxon classificationAnimaliaAraneaeSymphytognathidae

Y. Lin & S. Li sp. nov.

A6FE5C1D-4C69-5D37-BB53-F735FE6C79CD

http://zoobank.org/678D0283-8F7E-478A-A35E-EA51A2EA1026

Figs 30 , 31 , 38

##### Type material.

***Holotype*** ♂ (NHMSU Ar 127) and ***paratypes*** 2♀ (NHMSU Ar 128–129), **China**: Yunnan Province, Yiliang County, Jiuxiang Town, near entrance of Baiyan Cave, in low bush (25.15100°N, 103.40100°E; 1875 m), 24.VIII.2018, Y. Lin et al. leg.; 1♂ juvenile (NHMSU-HA141) and 1♀ juvenile (NHMSU-HA141) used for sequencing, GenBank: MT992017 and MT992016, same data as for preceding.

**Figure 30.**
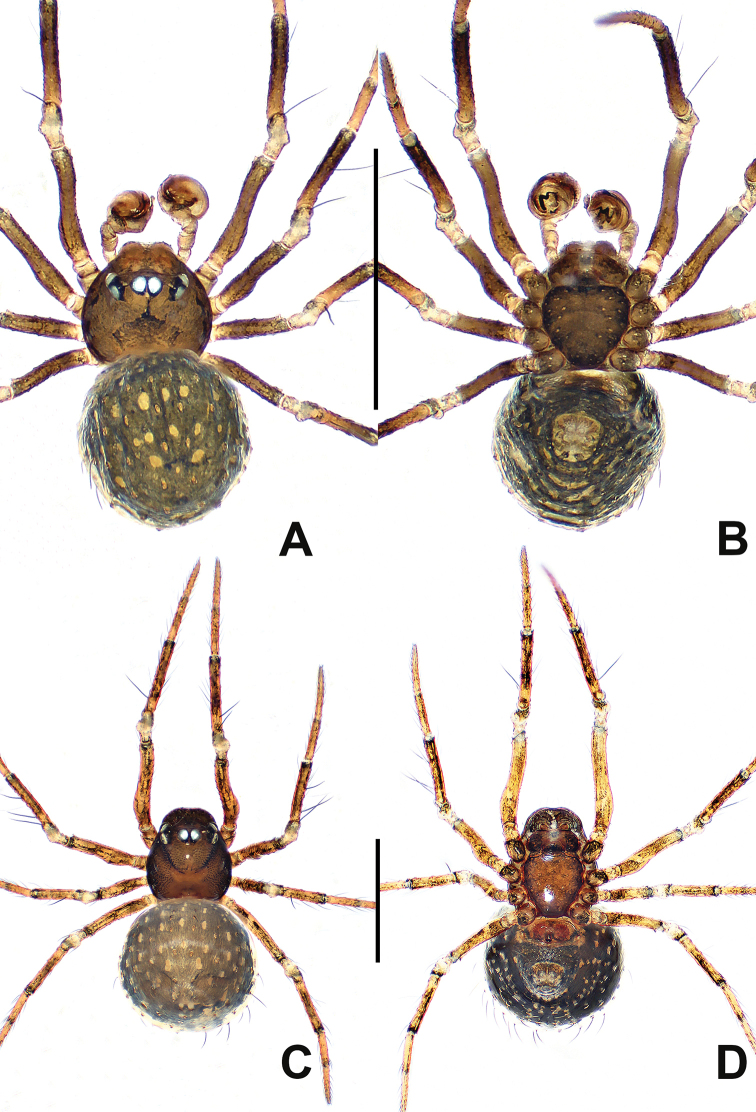
*Crassignatha si* sp. nov. **A** male habitus, dorsal, **B** male habitus, ventral **C** female habitus, dorsal **D** female habitus, ventral. Scale bars: 0.50 mm (**A–D**).

##### Diagnosis.

**Figure 31.**
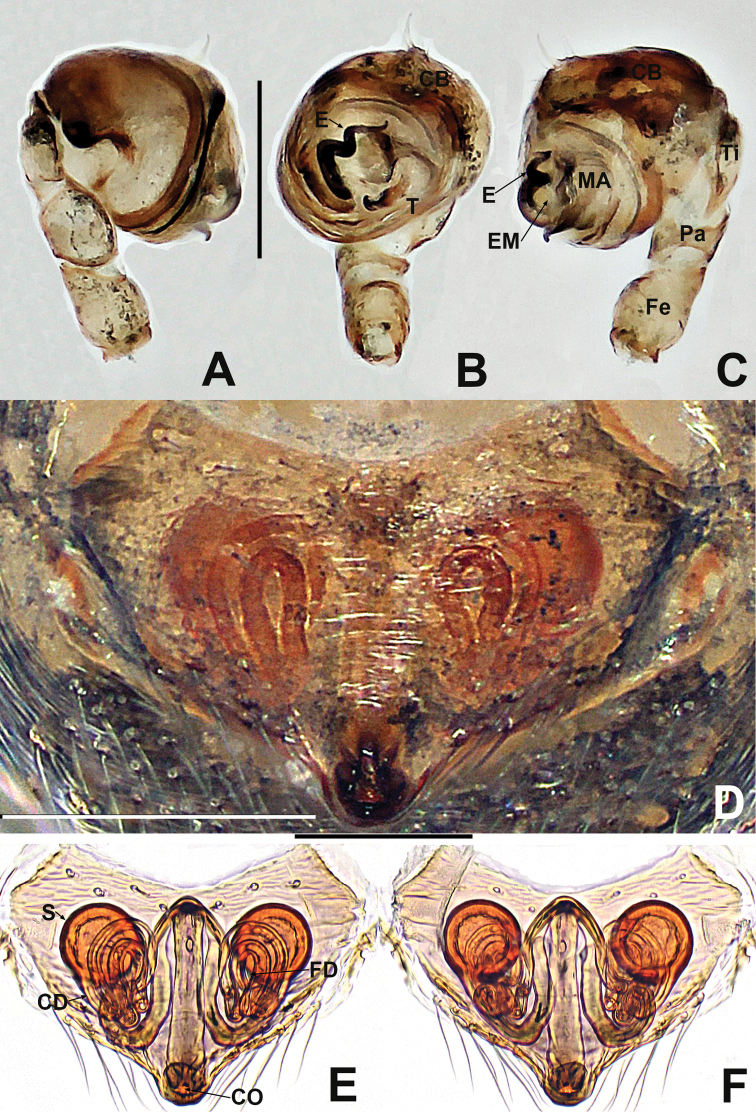
*Crassignatha si* sp. nov. **A** right male palp, prolateral **B** right male palp, ventral **C** right male palp, retrolateral **D** epigyne, ventral **E** vulva, ventral **F** vulva, dorsal. Scale bars: 0.10 mm (**A–F**).

The male of *C. si* sp. nov. differs from all other congeners by the presence of a helical embolus coiled slightly more than twice (Fig. 31B, C). The female of *C. si* sp. nov. is similar to *C. gudu* and *C. yamu* in the vulva configuration and the course of the copulatory ducts but can be distinguished from the latter two by the longer columnar atrium formed by the union of the copulatory ducts (Fig. 31F vs. Figs 11F, 35F).

##### Description.

**Male** (holotype). Total length 0.56. Carapace 0.20 long, 0.24 wide, 0.28 high. Clypeus 0.04 high. Sternum 0.16 long, 0.16 wide. Abdomen 0.36 long, 0.36 wide, 0.48 high. Length of legs: I 0.88 (0.24, 0.10, 0.20, 0.14, 0.20); II 0.76 (0.24, 0.08, 0.16, 0.10, 0.18); III 0.6 (0.18, 0.08, 0.12, 0.10, 0.12); IV 0.68 (0.18, 0.08, 0.14, 0.10, 0.18).

**Somatic characters** (Fig. 30A, B). ***Coloration***: prosoma and legs fuscous. Abdomen grayish green, ventrally darker than dorsally, with pale yellow speckles. ***Prosoma***: carapace nearly round, surface weakly sculptured. ALE protruded. PER slightly recurved. Cephalic area elevated. Clypeus concave. Chelicerae fused near base, covered with long setae anteriorly. Labium triangular. Sternum heart shaped, swollen, surface pitted. ***Legs***: femora and tibiae rough, granular. Tibia II with a clasping spur. ***Abdomen***: globose and rugose, lacking lateral scutum and circular plate. Spinnerets pale brown.

***Palp*** (Fig. 31A–C): femur swollen, wider than patella. Tibia flat. Cymbium weakly sclerotized, with a few setae distally. Cymbial tooth small, inconspicuous. Tegulum narrow, slightly swollen. Laminar median apophysis sub-rounded, translucent, with a small, hooked process on prolateral margin. Embolic membrane arises from behind median apophysis. Embolus long, helical, circling clockwise into almost two full coils.

**Female** (one of paratypes). Total length 0.92. Carapace 0.36 long, 0.32 wide, 0.36 high. Clypeus 0.06 high. Sternum 0.24 long, 0.24 wide. Abdomen 0.56 long, 0.56 wide, 0.52 high. Length of legs: I 1.22 (0.40, 0.16, 0.28, 0.18, 0.20); II 1.02 (0.28, 0.14, 0.22, 0.18, 0.20); III 0.66 (0.16, 0.08, 0.18, 0.10, 0.14); IV 0.86 (0.24, 0.08, 0.22, 0.14, 0.18).

**Somatic characters** (Fig. 30C, D). ***Coloration***: prosoma and legs dark brown. Abdomen color and modification as in male. ***Prosoma***: carapace nearly pear shaped, surface granular and sculptured, two strong setae medially. ALE slightly protruded. PER slightly recurved. Mouthparts as in male. Sternum scutiform, surface textured. ***Abdomen***: globose, with small sclerotized patches dorsally and ventrally. Spinnerets weakly sclerotized.

***Epigyne*** (Fig. 31D–F): epigynal area lightly sclerotized. Scape slightly protruded, as wide as long. Internal structures faintly visible via translucent tegument. Paired spermathecae separated by their diameter. Fertilization ducts slender, starting at the inside medial margin of spermathecae. Copulatory ducts long and extremely tortuous, connected to posterior margin of spermathecae, extending from under spermathecae to venter, forming two open loops, curving upward to center of vulva, then fusing into a columnar atrium that reaches copulatory opening at scape terminus.

##### Etymology.

The specific name is derived from the Chinese pinyin word for “spiral” (si), referring to the shape of the embolus, and is a noun in apposition.

##### Distribution.

China (Yunnan) (Fig. 38).

#### Crassignatha thamphra

Taxon classificationAnimaliaAraneaeSymphytognathidae

Y. Lin & S. Li sp. nov.

93B1FE40-F0E4-53C2-8CE7-E3722AD8F1A9

http://zoobank.org/AC2E340A-4A21-4B34-84F9-43F77A958B34

Figs 32 , 38

##### Type material.

***Holotype*** ♀ (IZCAS-Ar 41035), **Thailand**: Khon Kaen Province, Phu Pha Man District, Phu Pha Man Subdistrict, Tham Phra Cave (16.66603°N, 101.89623°E; 262 m), 10.XI.2016, H. Zhao, Y. Li and Z. Chen leg.; 1♀ (NHMSU-HA089) used for sequencing, GenBank: MT992003, same data as for preceding.

##### Diagnosis.

**Figure 32.**
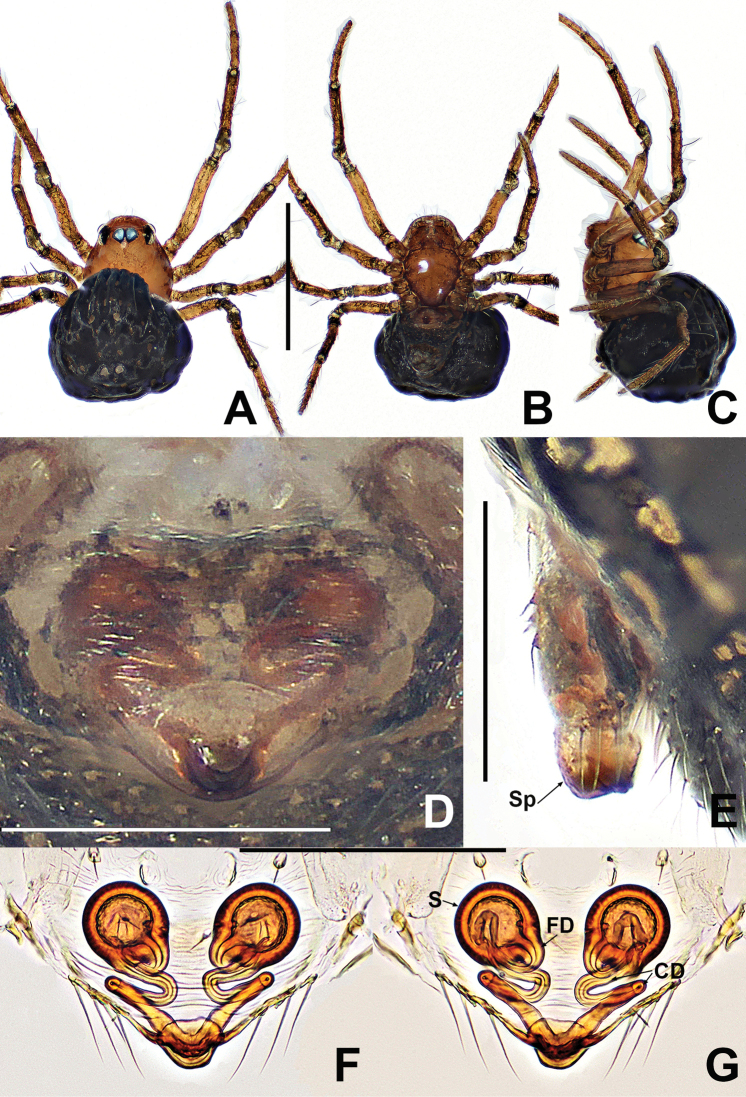
*Crassignatha thamphra* sp. nov. **A** female habitus, dorsal **B** female habitus, ventral **C** female habitus, lateral **D** epigyne, ventral **E** epigyne, lateral **F** vulva, ventral **G** vulva, dorsal. Scale bars: 0.50 mm (**A–C**); 0.10 mm (**D–G**).

This species differs other *Crassignatha* species, except *C. yinzhi*, by the copulatory ducts diagonally connected to the copulatory opening and not fused before reaching copulatory opening. It can be easily distinguished from *C. yinzhi* by the larger spermathecae separated by less than their diameter and the tighter turns of the copulatory ducts at center of the vulva (Fig. 32G vs. Fig. 37G).

##### Description.

**Female** (holotype). Total length 0.64. Carapace 0.32 long, 0.32 wide, 0.28 high. Clypeus 0.10 high. Sternum 0.20 long, 0.20 wide. Abdomen 0.44 long, 0.48 wide, 0.48 high. Length of legs: I 0.92 (0.28, 0.12, 0.20, 0.16, 0.16); II 0.74 (0.22, 0.10, 0.14, 0.12, 0.16); III 0.64 (0.16, 0.10, 0.12, 0.12, 0.14); IV 0.84 (0.24, 0.12, 0.18, 0.12, 0.18).

**Somatic characters** (Fig. 32A–C). ***Coloration***: prosoma yellowish brown. Legs dark brownish. Abdomen black with faint, sclerotized dots. ***Prosoma***: carapace nearly pear shaped, surface indistinctly textured. Cephalic area elevated. ALE slightly protruded. PER straight. Chelicerae bears sparse, short setae anteriorly. Labium tongue shaped. Sternum heart shaped, slightly plump, surface smooth, truncated posteriorly. ***Legs***: covered with setae and bristles. ***Abdomen***: abdominal shape irregular and surface rugose (caused by alcohol immersion). Spinnerets weakly sclerotized.

***Epigyne*** (Fig. 32D–G): epigynal area slightly sclerotized, bears a few long setae. Scape protruded, longer slightly than wide. Internal structures faintly visible via translucent tegument. Spermathecae separated by ~ ½ their diameter. Fertilization ducts thin, slender, originating from posteromedial margin of spermathecae, forming a U-shape. Copulatory ducts long, connected to the posterior surface of spermathecae, curving twice below spermathecae, linked diagonally, and fused to copulatory opening.

**Male.** Unknown.

##### Etymology.

The specific name is derived from the type locality; noun in apposition.

##### Distribution.

Thailand (Fig. 38).

#### Crassignatha xichou

Taxon classificationAnimaliaAraneaeSymphytognathidae

Y. Lin & S. Li sp. nov.

6F12036D-1325-56AF-BADE-957D216BD000

http://zoobank.org/41448EB7-E443-4F85-985A-556905296DA6

Figs 33 , 38

##### Type material.

***Holotype*** ♀ (NHMSU Ar 130), **China**: Yunnan Province, Wenshan Prefecture, Xichou County, near to radio and television transmitting tower, in leaf litter (23.43302°N, 104.67320°E; 1556 m), 17.V.2015, Z. Chen and Y. Li leg.; ***paratype*** 1♀ (NHMSU Ar 131), **China**: Yunnan Province, Nanjian County, Xiaowan Township, Huilongshan Village, near to entrance of Banpoyan Cave, in bushes (24.93353°N, 100.31443°E; 1990 m), 23.VIII.2018, Y. Lin et al. leg.

##### Diagnosis.

**Figure 33.**
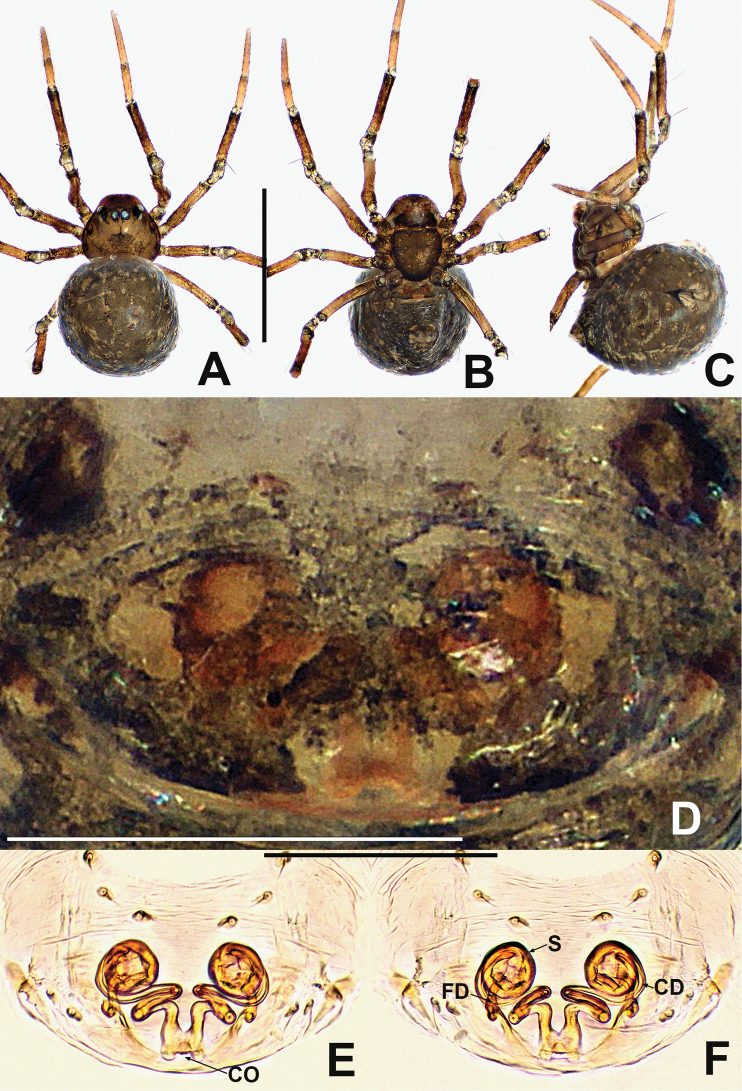
Female of *Crassignatha xichou* sp. nov. **A** habitus, dorsal **B** habitus, ventral **C** habitus, lateral **D** epigyne, ventral **E** vulva, ventral **F** vulva, dorsal. Scale bars: 0.50 mm (**A–C**); 0.10 mm (**D–F**).

This new species can be easily distinguished from all species of *Crassignatha* by the lack of a scape, the fertilization ducts starting at the posterolateral margin of the spermathecae, the copulatory ducts connecting to the anterolateral margin of spermathecae, fusing into an H-shaped atrium above copulatory opening (Fig. 33E, F).

##### Description.

**Female** (holotype). Total length 0.60. Carapace 0.24 long, 0.28 wide, 0.24 high. Clypeus 0.06 high. Sternum 0.16 long, 0.16 wide. Abdomen 0.44 long, 0.40 wide, 0.48 high. Length of legs: I 0.88 (0.24, 0.10, 0.20, 0.14, 0.20); II 0.78 (0.20, 0.10, 0.18, 0.12, 0.18); III 0.64 (0.14, 0.10, 0.14, 0.10, 0.16); IV 0.74 (0.20, 0.10, 0.16, 0.10, 0.18).

**Somatic characters** (Fig. 33A–C). ***Coloration***: carapace dark brownish, darker in thoracic center and margins. Mouthparts and sternum dark. Abdomen dark grayish. ***Prosoma***: carapace nearly pear shaped, surface indistinctly textured. Cephalic part raised. ALE slightly protruded. PER straight. Chelicerae lighter than endites and labium, bears short setae anteriorly. Labium nearly semicircular. Sternum scutiform, slightly swollen, surface pitted, truncated posteriorly. ***Legs***: metatarsi and tarsi yellow-brown, tibiae and femora dark. ***Abdomen***: oval dorsally, with light brown sclerotized dots. Spinnerets tiny.

***Epigyne*** (Fig. 33D–F): epigynal area dark, slightly sclerotized, with sparse, short setae. Scape absent. Internal structures faintly visible via translucent tegument. Paired spermathecae small, globose, separated by their diameter. Fertilization ducts originating posteromedially from spermathecae, coiled below spermathecae. Copulatory ducts long, connected to outer lateral margin of spermathecae, making four turns before merging into an H-shaped atrium.

**Male.** Unknown.

##### Etymology.

The specific name is derived from the type locality; noun in apposition.

##### Distribution.

China (Yunnan) (Fig. 38).

#### Crassignatha yamu

Taxon classificationAnimaliaAraneaeSymphytognathidae

Miller, Griswold & Yin, 2009

3D3ABA4D-D396-5353-8B19-9B8FCC9817BF

Figs 34 , 35 , 38

Crassignatha yamu Miller et al., 2009: 73, figs 76H, I, 86A–C, 87A, B (♂♀).

##### Type material.

***Holotype*** ♂ (HNU-CASENT 9029321) and ***paratype*** 1♀ (HNU-CASENT 9020752), **China**: Yunnan Province, Fugong County, S fork Yamu River, 1.51 km 150° SW of confluence [with N Fork], Gaoligongshan, moist shaded embankments (27.11905°N, 98.83108°E; 1723 m), 26.IV.2004, C. Griswold leg.; ***paratypes*** 2♀ (HNU-CASENT 9020736), **China**: Yunnan Province, Fugong County, 10^th^ km W NuJiang on Shibali Rd., N fork Yamu River, Gaoligongshan, moist earthen embankments (27.13795°N, 98.82240°E; 1850 m), 25.IV.2004, C. Griswold leg. Examined.

**Figure 34.**
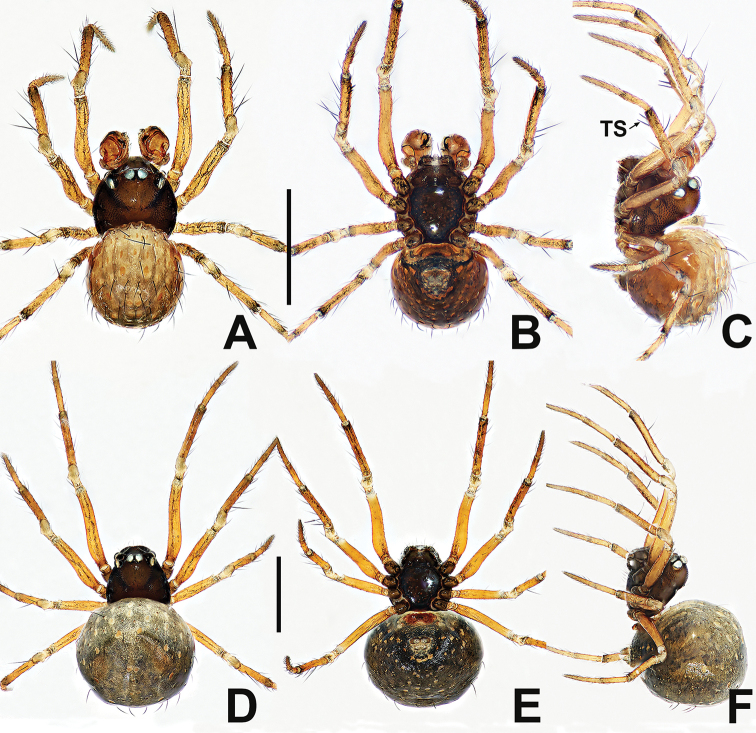
*Crassignatha yamu* Miller, Griswold & Yin, 2009 **A** male habitus, dorsal **B** male habitus, ventral **C** male habitus, lateral **D** female habitus, dorsal **E** female habitus, ventral **F** female habitus, lateral. Scale bars: 0.50 mm (**A–F**).

##### Other material examined.

6♂ 8♀ (NHMSU-HA115), **China**: Yunnan Province, Fugong County, Yamu River, ca. 1.51 km on 150° SW from confluence [with N fork], Gaoligongshan, moist shaded embankments (27.11905°N, 98.83108°E; 1723 m), 18.VIII.2018, Y. Lin et al.; 1♂ (NHMSU-HA115) and 1♀ (NHMSU-HA115) used for sequencing, GenBank: MT992011 and MT992010, same data as for preceding; 3♂ 7♀ (NHMSU-HA116), **China**: Yunnan Province, Fugong County, Shilajia Village, near estuary of Yamu River (27.13440°N, 98.82625°E; 1792 m), 18.VIII.2018, Y. Lin et al. leg.

##### Diagnosis.

**Figure 35.**
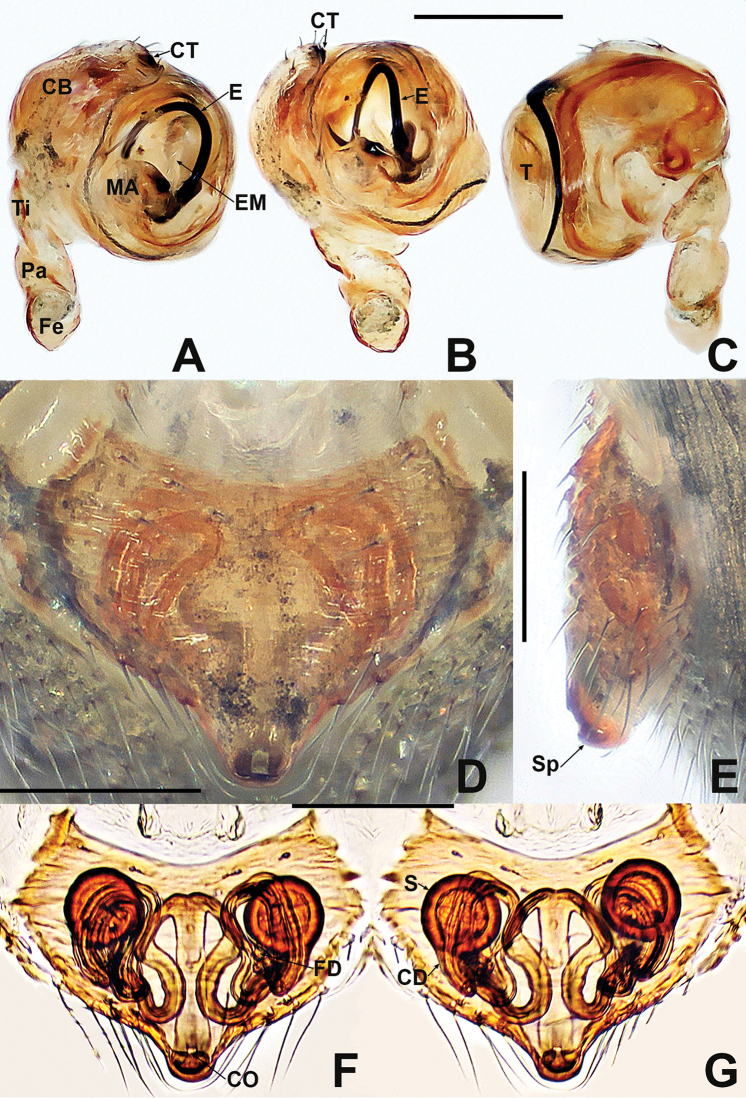
*Crassignatha yamu* Miller, Griswold & Yin, 2009 **A** male palp, prolateral **B** male palp, ventral **C** male palp, retrolateral **D** epigyne, ventral **E** epigyne, lateral **F** vulva, ventral **G** vulva, dorsal. Scale bars: 0.10 mm (**A–G**).

The male of *C. yamu* is most similar to that of *C. haeneli*, *C. danaugirangensis*, and *C. shiluensis* in the form of the palp and the long, linear embolus but differs from *C. haeneli* and *C. danaugirangensis* by the spiral embolus on the ventral portion of the palpal bulb (Fig. 25B vs. Wunderlich 1995: figs 18, 19; Miller et al. 2004: fig. 4); from *C. shiluensis* by having fewer loops of the embolus (Fig. 35B vs. Fig. 27A). The female is similar to that of *C. gudu* and *C. si* in vulva configuration and the course of the copulatory ducts but can be easily distinguished by having the columnar atrium longer than in *C. gudu* and shorter than in *C. si* (Fig. 35G vs. Figs 11F, 31F).

##### Description.

See Miller et al. (2009).

##### Distribution.

China (Yunnan) (Fig. 38).

#### Crassignatha yinzhi

Taxon classificationAnimaliaAraneaeSymphytognathidae

Miller, Griswold & Yin, 2009

46526C1A-AFAC-5AAD-ACDD-8E3FAC1F84D0

Figs 36 , 37 , 38

Crassignatha yinzhi Miller et al., 2009: 71, figs 76C, D, 78E, 79C, D, 80A–E, 81A, B, 82A–F (♂♀).

##### Type material.

***Holotype*** ♂ (HNU-CASENT 9029322) and ***paratypes*** 5♀ (HNU-CASENT 9022376), **China**: Yunnan Province, Longling County, Longjiang Township, Xiaoheishan Nature Reserve, 1.2 km SSE of Route S317 at 23.5 km, good primary broadleaf forest, dusting webs in understory (24.82888°N, 98.76001°E; 2020 m), 27–28.V.2005, C. Griswold leg.; 1♂ 1♀ (HNU-CASENT 9022354), **China**: Yunnan Province, Longling County, Longjiang Township, Xiaoheishan Nature Reserve, 1.2 km SSE of Route S317 at 23.5 km, good primary broadleaf forest, night collecting (24.82888°N, 98.76001°E; 2020 m), 28.V.2005, C. Griswold and D. Kavanaugh leg.; 1♀ (HNU-CASENT 9022396), **China**: Yunnan Province, Longling County, Longjiang Township, Xiaoheishan Nature Reserve, 1.2 km SSE of Route S317 at 23.5 km, good primary broadleaf forest, night collecting (24.82888°N, 98.76001°E; 2020 m), 26.V.2005, C. Griswold and D. Kavanaugh leg. Examined.

**Figure 36.**
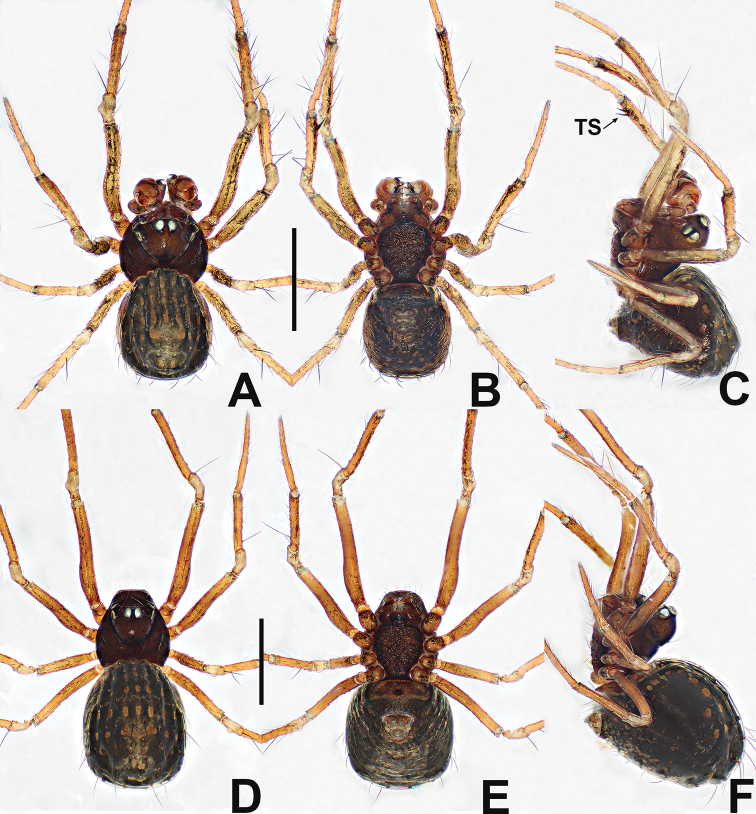
*Crassignatha yinzhi* Miller, Griswold & Yin, 2009 **A** male habitus, dorsal **B** male habitus, ventral **C** male habitus, lateral **D** female habitus, dorsal **E** female habitus, ventral **F** female habitus, lateral. Scale bars: 0.50 mm (**A–F**).

**Figure 37.**
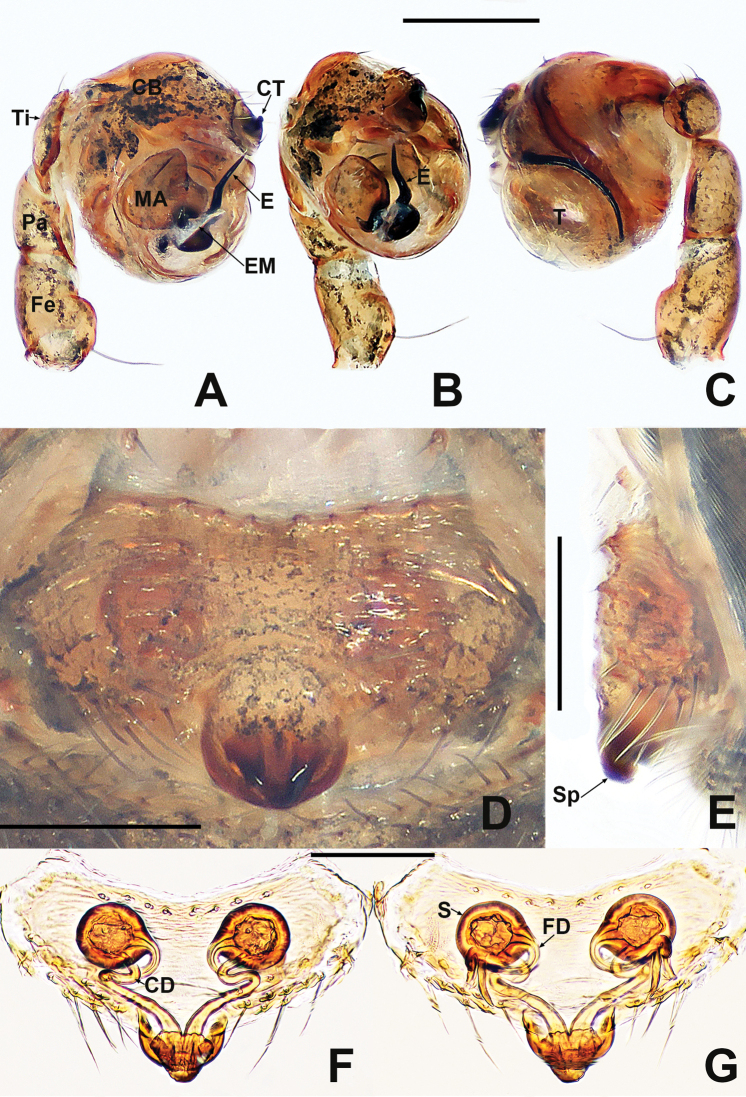
*Crassignatha yinzhi* Miller, Griswold & Yin, 2009 **A** male palp, prolateral **B** male palp, ventral **C** male palp, retrolateral **D** epigyne, ventral **E** epigyne, lateral **F** vulva, ventral **G** vulva, dorsal. Scale bars: 0.10 mm (**A–G**).

##### Other material examined.

6♂ 6♀ (NHMSU-HA117), **China**: Yunnan Province, Longling County, Longjiang Township, Xiaoheishan Nature Reserve (24.82888°N, 98.76001°E; 2020 m), 22.VIII.2018, Y. Lin et al. leg.; 1♂ (NHMSU-HA117) and 1♀ (NHMSU-HA117) used for sequencing, GenBank: MT992013 and MT992012, same data as for preceding; 1♂ 1♀ (NHMSU-HA127), **China**: Yunnan Province, Longling County, Longjiang Township, Xiaoheishan Nature Reserve, Gucheng Hill, good forest (24.82886°N, 98.75917°E; 2010 m), 22.VIII.2018, Y. Lin et al. leg.

##### Diagnosis.

The male of *C. yinzhi* is similar to *C. ertou* but can be distinguished by the nearly straight embolus and the caniniform cymbial tooth, rather than spiraled embolus and hooked cymbial tooth in the latter (Fig. 37A, B vs. Fig. 8A, B). The female of *C. yinzhi* is most similar to that of *C. thamphra* sp. nov. in vulva configuration but differs from the latter by the more widely separated spermathecae and the widely separated inflection points of the copulatory ducts (Fig. 37G vs. Fig. 32G).

##### Description.

See Miller et al. (2009).

##### Distribution.

**Figure 38.**
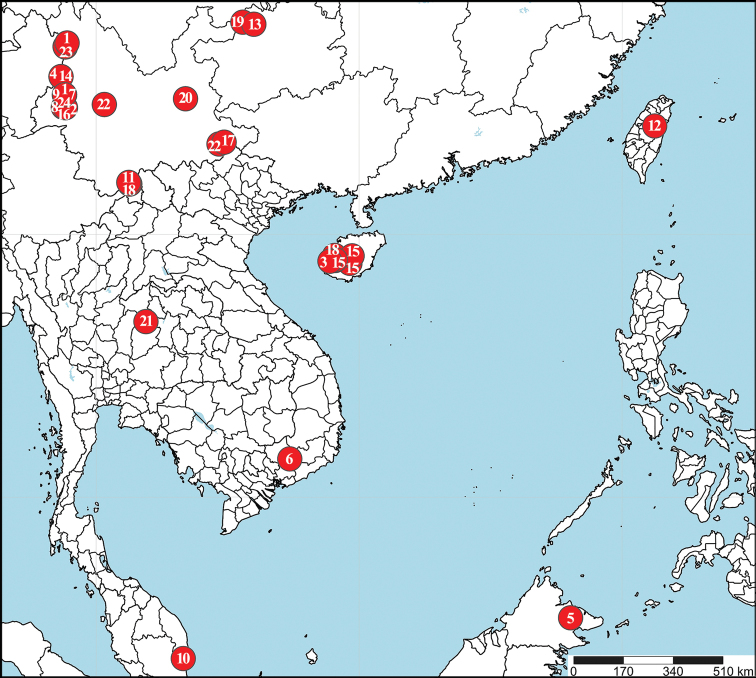
Distribution records of *Crassignatha* spp. in Asia. **1***C. baihua* sp. nov. **2***C. bangbie* sp. nov. **3***C. bicorniventris***4***C. changyan* sp. nov. **5***C. danaugirangensis***6***C. dongnai* sp. nov. **7***C. ertou***8***C. gucheng* sp. nov. **9***C. gudu***10***C. haeneli***11***C. mengla* sp. nov. **12***C. nantou* sp. nov. **13***C. nasalis* sp. nov. **14***C. pianma***15***C. quadriventris***16***C. quanqu***17***C. rostriformis* sp. nov. **18***C. shiluensis***19***C. shunani* sp. nov. **20***C. si* sp. nov. **21***C. thamphra* sp. nov. **22***C. xichou* sp. nov. **23***C. yamu***24***C. yinzhi*.

China (Yunnan) (Fig. 38).

## Supplementary Material

XML Treatment for Crassignatha

XML Treatment for Crassignatha baihua

XML Treatment for Crassignatha bangbie

XML Treatment for Crassignatha bicorniventris

XML Treatment for Crassignatha changyan

XML Treatment for Crassignatha dongnai

XML Treatment for Crassignatha ertou

XML Treatment for Crassignatha gucheng

XML Treatment for Crassignatha gudu

XML Treatment for Crassignatha mengla

XML Treatment for Crassignatha nantou

XML Treatment for Crassignatha nasalis

XML Treatment for Crassignatha pianma

XML Treatment for Crassignatha quadriventris

XML Treatment for Crassignatha quanqu

XML Treatment for Crassignatha rostriformis

XML Treatment for Crassignatha shiluensis

XML Treatment for Crassignatha shunani

XML Treatment for Crassignatha si

XML Treatment for Crassignatha thamphra

XML Treatment for Crassignatha xichou

XML Treatment for Crassignatha yamu

XML Treatment for Crassignatha yinzhi

## Appendix 1

**Table A1.** Uncorrected genetic pairwise distance (below diagonal) and standard error (above diagonal) of a partial fragment of COI from the seventeen species discussed in this text.

	Species		1		2	3	4	5	6		7		8		9		10	11		12		13		14		15	16		17	
			♀	♂	♀	♀	♀	♀	♀	♂J	♀	♂	♀	♂	♀	♂	♀	♀	♂	♀	♂J	♀	♂J	♀J	♂J	♀	♀	♂	♀	♂
1	*C. baihua* sp. nov.	♀		0.003	0.012	0.008	0.013	0.005	0.008	0.008	0.012	0.012	0.012	0.012	0.011	0.011	0.007	0.010	0.011	0.016	0.016	0.013	0.013	0.012	0.012	0.012	0.012	0.012	0.012	0.012
		♂	0.005		0.012	0.008	0.013	0.005	0.008	0.008	0.012	0.012	0.012	0.012	0.011	0.010	0.007	0.010	0.010	0.016	0.016	0.013	0.013	0.011	0.011	0.012	0.012	0.011	0.012	0.012
2	*C. bangbie* sp. nov.	♀	0.090	0.088		0.012	0.013	0.012	0.011	0.011	0.012	0.012	0.011	0.011	0.010	0.010	0.012	0.010	0.011	0.015	0.015	0.012	0.011	0.011	0.011	0.013	0.012	0.012	0.012	0.012
3	*C. dongnai* sp. nov.	♀	0.045	0.044	0.090		0.013	0.009	0.007	0.007	0.012	0.012	0.012	0.011	0.011	0.011	0.008	0.011	0.011	0.016	0.016	0.013	0.013	0.012	0.012	0.012	0.012	0.012	0.012	0.012
4	*C. ertou*	♀	0.104	0.099	0.101	0.106		0.013	0.013	0.013	0.012	0.012	0.013	0.013	0.013	0.013	0.013	0.012	0.012	0.017	0.017	0.013	0.013	0.013	0.013	0.013	0.013	0.013	0.011	0.011
5	*C. gucheng* sp. nov.	♀	0.022	0.021	0.087	0.044	0.103		0.008	0.008	0.012	0.012	0.012	0.012	0.011	0.011	0.006	0.011	0.011	0.016	0.016	0.012	0.012	0.011	0.011	0.012	0.012	0.011	0.012	0.012
6	*C. mengla* sp. nov.	♀	0.040	0.039	0.080	0.026	0.103	0.037		0.000	0.011	0.011	0.011	0.011	0.011	0.011	0.007	0.010	0.010	0.016	0.016	0.012	0.012	0.011	0.011	0.012	0.012	0.011	0.012	0.012
		♂J	0.040	0.039	0.080	0.026	0.103	0.037	0.000		0.011	0.011	0.011	0.011	0.011	0.011	0.007	0.010	0.010	0.016	0.016	0.012	0.012	0.011	0.011	0.012	0.012	0.011	0.012	0.012
7	*C. nantou* sp. nov.	♀	0.097	0.096	0.090	0.094	0.101	0.097	0.090	0.090		0.000	0.010	0.010	0.012	0.012	0.012	0.011	0.012	0.016	0.016	0.008	0.008	0.012	0.012	0.013	0.013	0.012	0.012	0.012
		♂	0.097	0.096	0.090	0.094	0.101	0.097	0.090	0.090	0.000		0.010	0.010	0.012	0.012	0.012	0.011	0.012	0.016	0.016	0.008	0.008	0.012	0.012	0.013	0.013	0.012	0.012	0.012
8	*C. nasalis* sp. nov.	♀	0.088	0.087	0.081	0.085	0.097	0.080	0.085	0.085	0.071	0.071		0.004	0.011	0.011	0.012	0.011	0.011	0.015	0.015	0.010	0.010	0.011	0.011	0.012	0.012	0.011	0.011	0.011
		♂	0.083	0.081	0.078	0.080	0.096	0.078	0.080	0.080	0.069	0.069	0.010		0.011	0.011	0.011	0.011	0.011	0.015	0.015	0.010	0.011	0.011	0.011	0.012	0.012	0.011	0.011	0.011
9	*C. pianma*	♀	0.083	0.081	0.062	0.085	0.105	0.076	0.083	0.083	0.105	0.105	0.085	0.085		0.003	0.011	0.011	0.011	0.016	0.016	0.011	0.012	0.011	0.011	0.013	0.013	0.012	0.011	0.011
		♂	0.080	0.078	0.057	0.083	0.099	0.076	0.080	0.080	0.101	0.101	0.081	0.081	0.005		0.011	0.010	0.011	0.016	0.016	0.011	0.011	0.011	0.011	0.013	0.012	0.012	0.011	0.011
10	*C. quadriventris*	♀	0.032	0.031	0.088	0.044	0.104	0.027	0.035	0.035	0.101	0.101	0.088	0.083	0.081	0.078		0.010	0.011	0.016	0.016	0.012	0.012	0.010	0.010	0.012	0.012	0.011	0.012	0.012
11	*C. rostriformis* sp. nov.	♀	0.068	0.066	0.071	0.074	0.094	0.071	0.066	0.066	0.088	0.088	0.074	0.071	0.073	0.067	0.068		0.001	0.016	0.016	0.012	0.012	0.010	0.010	0.011	0.011	0.010	0.012	0.011
		♂	0.069	0.068	0.073	0.076	0.096	0.073	0.067	0.067	0.090	0.090	0.076	0.073	0.074	0.069	0.069	0.002		0.016	0.016	0.012	0.012	0.010	0.010	0.011	0.011	0.010	0.012	0.011
12	*C. shiluensis*	♀	0.144	0.150	0.155	0.147	0.175	0.147	0.151	0.151	0.153	0.153	0.140	0.136	0.157	0.155	0.149	0.155	0.155		0.000	0.015	0.015	0.015	0.015	0.015	0.017	0.016	0.016	0.016
		♂J	0.144	0.150	0.155	0.147	0.175	0.147	0.151	0.151	0.153	0.153	0.140	0.136	0.157	0.155	0.149	0.155	0.155	0.000		0.015	0.015	0.015	0.015	0.015	0.017	0.016	0.016	0.016
13	*C. shunani* sp. nov.	♀	0.099	0.097	0.088	0.101	0.108	0.092	0.094	0.094	0.041	0.041	0.068	0.071	0.096	0.092	0.092	0.092	0.094	0.134	0.134		0.004	0.011	0.011	0.013	0.013	0.012	0.011	0.012
		♂J	0.101	0.099	0.087	0.103	0.110	0.094	0.096	0.096	0.042	0.042	0.069	0.073	0.097	0.094	0.094	0.094	0.096	0.142	0.142	0.010		0.011	0.011	0.013	0.013	0.012	0.012	0.012
14	*C. si* sp. nov.	♀J	0.088	0.087	0.078	0.083	0.094	0.081	0.081	0.081	0.097	0.097	0.080	0.078	0.083	0.078	0.074	0.064	0.066	0.149	0.149	0.088	0.087		0.000	0.013	0.007	0.006	0.011	0.011
		♂J	0.088	0.087	0.078	0.083	0.094	0.081	0.081	0.081	0.097	0.097	0.080	0.078	0.083	0.078	0.074	0.064	0.066	0.149	0.149	0.088	0.087	0.000		0.013	0.007	0.006	0.011	0.011
15	*C. thamphra* sp. nov.	♀	0.090	0.088	0.094	0.094	0.104	0.083	0.094	0.094	0.108	0.108	0.088	0.087	0.095	0.092	0.088	0.080	0.081	0.142	0.142	0.104	0.110	0.095	0.095		0.013	0.012	0.011	0.011
16	*C. yamu*	♀	0.096	0.096	0.096	0.101	0.101	0.085	0.090	0.090	0.109	0.109	0.087	0.083	0.103	0.097	0.090	0.076	0.078	0.163	0.163	0.101	0.103	0.036	0.036	0.097		0.003	0.012	0.012
		♂	0.094	0.092	0.087	0.097	0.096	0.081	0.087	0.087	0.099	0.099	0.082	0.078	0.094	0.088	0.087	0.071	0.073	0.155	0.155	0.092	0.094	0.027	0.027	0.094	0.008		0.011	0.011
17	*C. yinzhi*	♀	0.092	0.090	0.090	0.088	0.076	0.081	0.087	0.087	0.097	0.097	0.074	0.069	0.083	0.081	0.087	0.081	0.083	0.151	0.151	0.083	0.088	0.083	0.083	0.080	0.094	0.088		0.004
		♂	0.092	0.090	0.090	0.088	0.073	0.081	0.087	0.087	0.097	0.097	0.071	0.069	0.080	0.078	0.087	0.074	0.076	0.153	0.153	0.088	0.090	0.080	0.080	0.078	0.094	0.088	0.010	
